# Combination of structure-based virtual screening, molecular docking and molecular dynamics approaches for the discovery of anti-prion fibril flavonoids

**DOI:** 10.3389/fmolb.2022.1088733

**Published:** 2023-01-05

**Authors:** Cheng-Ping Jheng, Cheng-I Lee

**Affiliations:** ^1^ Department of Biomedical Sciences, National Chung Cheng University, Chia-Yi, Taiwan; ^2^ Center for Nano Bio-Detections, National Chung Cheng University, Chia-Yi, Taiwan; ^3^ Center for Innovative Research on Aging Society (CIRAS), National Chung Cheng University, Chia-Yi, Taiwan

**Keywords:** flavonoid, quercetin, prion fibril, docking, molecular dynamics

## Abstract

Prion diseases are a group of rare neurodegenerative diseases caused by the structural conversion of cellular prion into Scrapie prion resulting aggregated fibrils. Therapy of prion diseases has been developed for several decades, especially drug designs based on the structure of prion monomers. Unfortunately, none of the designed anti-prion drugs function well clinically. To fight against prion fibrils, a drug design based on the precise structure of mammalian prion fibrils is highly required. Fortunately, based on the advantage of newly advanced cryo-electron microscopy (cryo-EM) in the deconvolution of large complexes, three prion fibril structures were resolved in the last 2 years. Based on the cryo-EM solved prion fibril structures, we are able to find some molecules fighting against prion fibrils. Quercetin, one flavonoid molecule in the polyphenol group, has been found to disaggregate the prion fibrils *in vitro*. In this study, we performed the molecular docking and molecular dynamics simulation on quercetin-like molecules possessing pharmacological properties to evaluate the anti-prion ability of tested molecules. As a result, four quercetin-like molecules interact with prion fibril and decrease the β-strand content by converting some β-strands into loop and helical structures to disintegrate the existing fibril structure. The results of this study are significant in the treatment of prion diseases, and the approaches used in this study are applicable to other amyloid diseases.

## 1 Introduction

Transmissible spongiform encephalopathies (TSEs) or prion diseases are a group of rare neurodegenerative diseases. Uniquely, prion diseases occur familial, sporadic and infectious forms (Gambetti et al., 2003). According to the statistics, over eighty percent of clinical cases are recognized as sporadic (Appleby et al., 2009; Sun et al., 2020). Prion diseases are ascribed to misfolding of prion protein. Structurally, prion protein has two distinct isoforms such as Cellular form prion protein (PrP^C^) and Scripe form prion protein (PrP^Sc^). PrP^C^ is structurally rich with α-helix and is anchored to membranes by glycosyl phosphatidyl inositol (GPI). Under unknown conditions, PrP^C^ structurally converts into an infectious isoform—PrP^Sc^ with high β-sheet conformation. PrP^C^ is sensitive to proteinase K (PK), whereas PrP^Sc^ is resistant to PK. The existing PrP^Sc^ induces more PrP^C^ to replicate into PrP^Sc^. Furthermore, PrP^Sc^ initiates a subsequent cascade of structural conversion to form insoluble amyloid PrP fibrils with abundant cross-β-sheets. The PrP^Sc^ spreads from the central nervous system (CNS) to other tissues *via* peripheral nervous system (PNS) (Gough and Maddison, 2010).

The misfolded PrP recruits the native PrP^C^ for propagation, and then expands the transmission region in the brain (Prusiner, 1998). In *in vitro* model and *in vivo* mouse model, PrP^Sc^ induces α-synuclein fibril formation and α-synuclein fibril also converts PrP^C^ to PrP^Sc^ (Masliah et al., 2012; Katorcha et al., 2017). Prion infection induces the decrease of α-synuclein expression and the hyperphosphorylation of p(S129)-α-synuclein, one hallmark of Parkinson’s disease, is colocalized with PrP aggregates (Chen et al., 2021). In the case of abnormal β-amyloid (Aβ) amyloidosis, infected prion patients or mice had Aβ deposition in brain without tau-pathology (Cali et al., 2018; Ezpeleta et al., 2019; Jankovska et al., 2021). In PrP^Sc^-infected APP23 mice, the downregulation of the PDK1-TACE pathway triggers the pathway into beta-site amyloid precursor protein cleaving enzyme (BACE) cleavage, which produces Aβ40/42 and induces Aβ multimer formation, decreasing the survival rate (Ezpeleta et al., 2019). Shortly speaking, misfolding of PrP is not only the cause of prion diseases, but also the inducer of other diseases. Inhibiting the progression of PrP^Sc^ expansion in neurodegenerative diseases is a critical issue.

Scientists have been working on the development of anti-prion agents for decades (Gandini and Bolognesi, 2017). The function of an anti-prion agent includes inhibiting PrP^C^-PrP^Sc^ conversion, inhibiting PrP^Sc^ polymerization, and promoting fibril degradation (Uliassi et al., 2022). It is difficult to interrupt the fibrillation once the protein misfolding is initiated. Therefore, many studies are focused on blocking the structural transition of amyloid protein into amyloid fibril. For prion diseases, the most common strategy is to stabilize the structure of PrP^C^ with chemical compounds. Under this strategy, the structure of PrP^C^ is critical. Several PrP^C^ structures have been solved by nuclear magnetic resonance spectroscopy (NMR) (Riek et al., 1996; Zahn et al., 2000). Some structure-based drug designs were conducted based on these NMR structures (Baral et al., 2014; Herrmann et al., 2015; Ishibashi et al., 2016). Subsequently, several anti-prion compounds, e.g. promazine, phenothiazine derivatives, and polythiophenes derivates were found effective in pharmacological studies (Baral et al., 2014; Herrmann et al., 2015; Ishibashi et al., 2016). However, these anti-prion compounds failed in clinical trials. These failures might be ascribed to the improper receptors (mammalian PrP^C^ protein monomers or yeast prion monomers) of these anti-prion compounds. It is considered to change the drug target of structure-based design from monomeric PrP to the structurally distinct mammalian PrP fibrils. This is the approach used in this study.

Solving the molecular structure of amyloid fibrils is very challenging. Fortunately, with the advantages of a revolutionary technique called cryo-EM, the three-dimensional structures of biological macromolecules with high resolution can be solved in a short time (Callaway, 2020). In 2020–2021, three structures of wild-type prion fibrils were solved by cryo-EM at a resolution within 3.5 Å, including fibrils of PrP_106–145_ (fPrP_106–145_, PDB 6UUR) converted with 4 M urea at pH 4.0 (Glynn et al., 2020), fibrils of PrP_170–229_ (fPrP_170–229_, PDB 6LNI) with 2 M guanidinium chloride (Gdn-HCl) at pH 7.4 (Wang et al., 2020) and fibrils of PrP_95–227_ (fPrP_95–227_, PDB 7LNA) from 263K mouse brain homogenate (Kraus et al., 2021). The cross-β-sheets and high β-sheet content from three identified prion amyloid structures are consistent with the early report (Wille et al., 2002). The high-resolution structure of fibrils can be used to resolve the interaction network contributing to fibril stability and can be used to design drugs for weakening the interaction to disrupt protein fibrils.

Polyphenols are a large family of natural compounds commonly found in plants. They are generally composed of multiple phenol units and are moderately water-soluble. Polyphenol molecules are categorized into five groups: phenolic acids, flavonoids, stilbenes, tannins, and lignans (Tijjani et al., 2020). It is found that polyphenols decrease fibril by inhibiting fibril formation or by fibril disaggregation with specific pathways depending on the proteins in Alzheimer’s and Parkinson’s diseases (Freyssin et al., 2018). In addition, these polyphenols serve as antioxidants in the cells to detoxify the oxidation of lipid, protein, and DNA from oxidative stress. The imbalance of production and scavenge of reactive oxygen species (ROS) or reactive nitrogen species (RNS) induces serious respond correlated to neurodegenerative diseases (Bhat et al., 2015). Quercetin, a kind of flavonoid, is absorbed by the small intestinal mucosa and then transported through blood vessels into the liver (Ulusoy and Sanlier, 2020). Quercetin is commonly found in our diet and has many benefits for anti-inflammation, anti-cancer, anti-neurological diseases, and diabetes mellitus (Ulusoy and Sanlier, 2020). Recently, we found that quercetin disaggregates mature prion fibrils, accelerates fibril clearance with PK, and reduces ROS levels in Neuro-2a cells (Yu and Lee, 2020). In addition, quercetin binding accelerates the formation of PK-sensitive and structure-less fibrils (Yu et al., 2022).

In drug development, structure-based drug design is the mainstream compared to ligand-based pharmacophore (Sharma et al., 2021). Structure-based drug design has resulted in the FDA’s approval of human immunodeficiency virus-1 protease inhibitors for acquired immunodeficiency syndrome, isoniazid for tuberculosis, and Pim-1 kinase inhibitors for cancer (Wlodawer and Vondrasek, 1998; Batool et al., 2019). A computer-aided drug design searches for the drug candidates that includes molecular docking and molecular dynamics (MD) simulation in a time saving way (Śledź and Caflisch, 2018). The protein-ligand interaction at the atomic level is resolved by MD simulation, and it is a complementary tool to NMR spectroscopy and X-ray crystallography.

**FIGURE 1:**
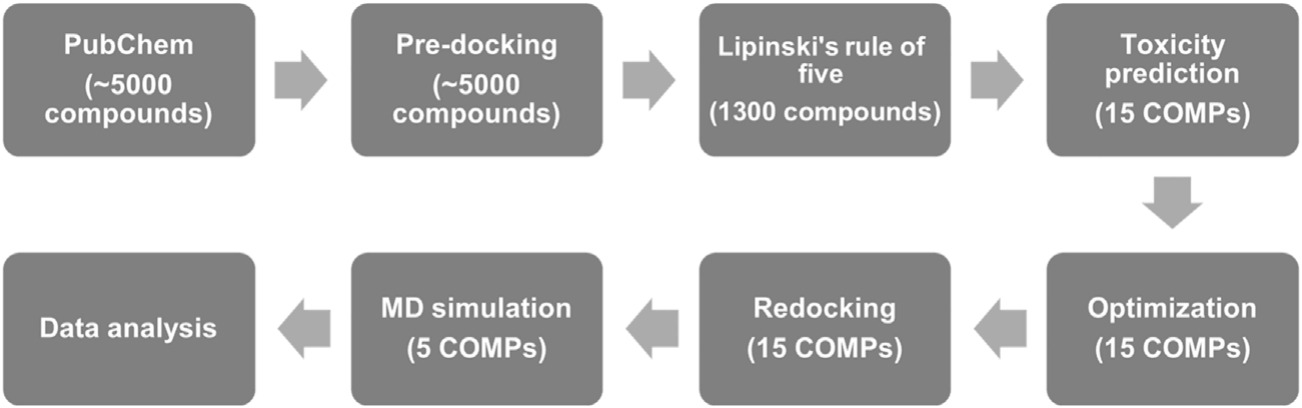
The flow chart of anti-prion screening based on pharmaceutical activity prediction, molecular docking, and MD simulation.

In this study, we used the structure of fPrP_170–229_ as the target for the screening of quercetin-like molecules in a pre-docking. To largely reduce the computing time, Lipinski’s rule of five (Lipinski, 2004) was applied to screen orally administrated and lipophilic drug candidates with pharmaceutical activity for curing the prion diseases in CNS, PNS, and other tissues. Based on the docking score and toxicity prediction, we minimized the number of target compounds from 1,300 compounds to 15 compounds (15 COMPs). The selective candidates were further evaluated for their toxicity and sequentially computed as shown in Figure 1.

## 2 Methods

### 2.1 Calculation of Debye–Waller factor (*B*-factor) for fPrP_170-229_

The Debye–Waller factor (*B*-factor) was calculated by AmberTools17 (Case et al., 2017) for the comparison of the modeled structures and the cryo-EM structure. The *B*-factor is calculated as the following equation,

$$B=\frac{8\pi^{2}}{3}\langle u^{2}\rangle$$ (1)

where, u is the displacement of scattering center. The *B*-factor was further normalized as following,

$$normalized\;B-factor\;\left(B^{\prime }\right)=\frac{B-\bar{B}}{\sigma_{B}}$$ (2)

where, $\bar{B}$ and $\sigma_{B}$ represent the average of *B*-factor and standard deviation of *B*-factor, respectively (Carugo and Argos, 1997).

### 2.2 Pre-docking of quercetin-like polyphenol molecules

For the potential ligands of fPrP_170–229_, we have chosen ∼5,000 polyphenols with >80% similarity to quercetin from PubChem (Kim et al., 2016). Prior to pre-docking, these polyphenol molecules were structurally optimized with universal force field (uff) (Rappe et al., 1992) by PyRx software (Dallakyan and Olson, 2015). The initial structure of fPrP_170–229_ (Wang et al., 2020) was protonated at pH 7.3 using H++ automatic prediction (Gordon et al., 2005). PyRx coupled with AutoDock Vina (Trott and Olson, 2010) was used for high-throughput screening of protein-ligand pre-docking. In this pre-docking, the global searching exhaustiveness of 30 was applied for the balance between accuracy and computing time (Forli et al., 2016).

### 2.3 Selection of drug candidates with high pharmaceutical activity

To reduce the number of ligand molecules for further computing, Lipinski’s rule of five was applied to molecules with a high docking score in pre-docking (1,300 molecules in total). The top 100 molecules with more negative docking score are listed in Supplementary Table S1, in order to find out the best 15 COMPs (COMP **1** ∼ COMP **15**) for the following search. The considered properties of ligand molecules include: less than five hydrogen bond donors, less than ten hydrogen bond acceptors, molecular mass less than 500 Dalton, and the partition coefficient (logP) less than 5. Furthermore, the pharmacokinetics and toxicity were predicted by ProToxII web server (Banerjee et al., 2018) and Molinspiration property calculation service (Ulica, 2021).

### 2.4 Structure optimization of COMPs in water

According to the induce fit theory, the molecule has its structure in a water solution and then adjusts the conformation to form the complex with protein. To fit this model, we optimized our small molecules in the water model before redocking. Based on the screening result of Lipinski’s rule of five, quercetin and 15 COMPs were structurally optimized by B3LYP (Lee et al., 1988) with basis set 631G++(3dp, 3df) (Petersson and Al-Laham, 1991) and solvated in water with a Polarizable Continuum model (PCM, dielectric constant = 78.33553) (Pascual-ahuir et al., 1994) using Gaussian 09 (Frisch et al., 2016).

### 2.5 Redocking of compounds with fibrils

Quercetin and 15 structurally optimized COMPs were individually set as ligands for molecular docking. The PyRx, coupled with AutoDock Vina, was used for the automatic docking of COMP-fPrP_170–229_. The exhaustiveness is set as 30. The dimension of grid box for search space was 174.71 × 67.513 × 31.106 (X*Y*Z) Å^3^. The grid box was centered at the geometry center of fPrP_170–229_ (X = 202.924, Y = 202.931, and Z = 230.919).

### 2.6 MD simulation

Primarily, we performed both implicit and explicit models for MD simulation of fPrP_170–229_ to test the reliability of implicit solvent model. In explicit solvent model with periodic boundary conditions, fPrP_170–229_ was solvated in a truncated octahedral water box including 75,862 transferable intermolecular potential 3P (TIP3P) water molecules and neutralized with eighteen sodium counter cations. In implicit solvent model, the simulations were conducted with modified generalized born model (GB, igb = 5) (Onufriev et al., 2004) containing 0.2 M salt concentration. Based on good agreement of 40 ns simulation results from these two solvation models, the rest of MD simulation was performed with implicit solvent model. To test the stability of quercetin-fPrP_170–229_ and COMP-fPrP_170–229_ complexes, we selected quercetin and the top five COMPs with the strongest binding affinity from AutoDock Vina to perform MD simulation with Amber 17 (Case et al., 2017) using force field Amber14SB (Maier et al., 2015) for 162 ns. According to our re-docking result, the top three strong bindings of COMP-fPrP_170–229_ complexes were considered as the initial structures of models A, B, and C, respectively, for each COMP. Three models were performed independently using MD simulation.

### 2.7 Evaluation of the binding energy between the COMP and fPrP_170–229_

After MD simulation of quercetin-fPrP_170–229_ and COMP-fPrP_170–229_ complexes, the binding energy of ligand-fPrP_170–229_ complexes was analyzed by AmberTools17 with molecular mechanics/generalized Born surface area (MM/GBSA) during the last 1ns of MD simulation (Miller et al., 2012; Case et al., 2017). PBRadii mbondi2 and force field Amber14SB were applied in the calculation using Amber17. The enthalpy in molecular mechanism in standard state ( $\Delta H_{MM}^{0}$ ) is calculated as the equation shown below.

$$\Delta H_{MM}^{0}=E_{vdW}^{0}+E_{EEL}^{0}$$ (3)

Where, $\Delta H_{MM}^{0}$ is contributed by van der Waals force ( $E_{vdW}^{0}$ ) and electrostatic interaction ( $E_{EEL}^{0}).$

### 2.8 Analysis of MD results

After the docking and MD simulation, the quercetin and COMP binding sites were analyzed by LigPlot+ (Wallace et al., 1995) and visualized in the binding cavity by PyMol (Schrodinger, 2015). The structures of the COMP-fPrP_170–229_ complexes were visualized by visual molecular dynamics (Humphrey et al., 1996). Ten β-sheets in fPrP_170–229_ are labelled alphabetically for visualization. The dictionary of protein secondary structure (DSSP) algorithm (Kabsch and Sander, 1983) in AmberTools17 was used for assigning protein secondary structure into eight types through estimating the energy of hydrogen bonds.

### 2.9 Calculation of root mean square fluctuation (RMSF) and root mean square deviation (RMSD)

The fluctuation and the movement of fPrP_170–229_ residues in MD simulation were examined by RMSF and RMSD, respectively. The RMSF and RMSD were calculated by AmberTools17.

## 3 Results

### 3.1 Calibration of the force-field

**FIGURE 2:**
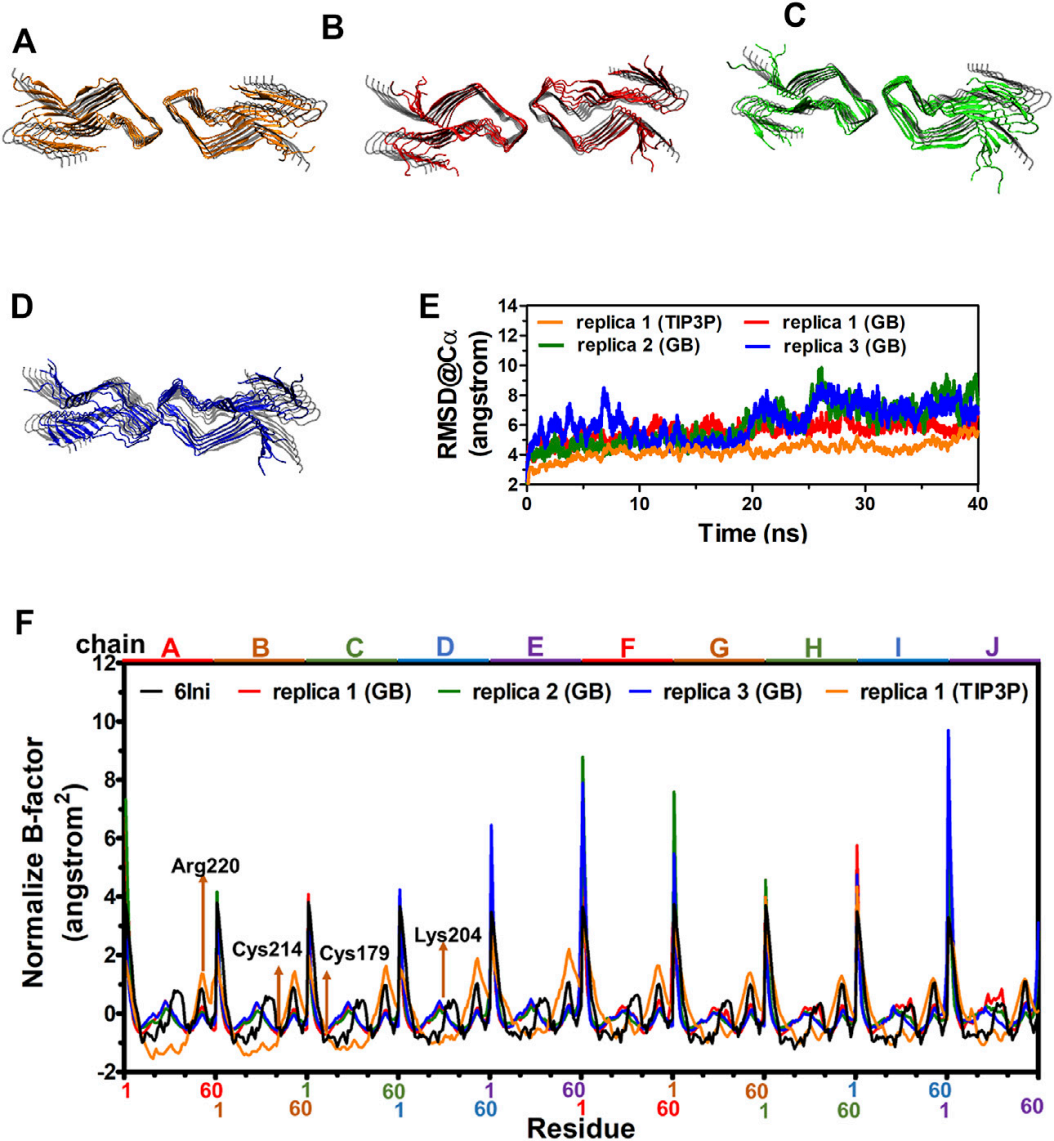
MD structures of fPrP_170–229_ selected from the most populated clusters from three independent replica from explicit solvent model and implicit solvent model shown in **(A)** and **(B**–**D)**, respectively. The cryo-EM structure of fPrP_170–229_ is shown in gray as a comparison. **(E)** The RMSD of fPrP_170–229_ backbone in replica 1 from explicit solvent (TIP3P) and replica 1–3 from implicit solvent (GB). **(F)** Normalized *B*-factor from three fPrP_170–229_ replica in implicit solvent and one replica in explicit solvent.

The cryo-EM structure of fPrP_170–229_ is composed of 10 repeated β-sheets from residues 170–229 (Wang et al., 2020). For MD simulations to be realistic, the solvation model is essential. Depending on the application, the results of the implicit solvent model sometimes agree with the conclusions of the explicit solvent model and experimental observation (Huang and Stultz, 2007). Two 40 ns-MD simulations of two solvent models were carried out for comparison in order to ensure the applicability of the implicit solvent model in the simulation of the fPrP_170–229_ structure. Figure 2 compares the most populated cluster from four independent simulations of two solvent models (Figure 2A for an explicit solvent; Figures 2B–D for an implicit solvent). The simulated fPrP_170–229_ backbone’s RMSD from the cryo-EM structure is constant at 5–9 Å for implicit solvent models and 4–5 Å for explicit solvent models, respectively (Figure 2E). The flexibility of the normalized *B*-factor (B′) of the simulated structure and the cryo-EM structure is compared (Figure 2F). The profile of B′ in the cryo-EM fPrP_170–229_ structure exhibits a periodic repeat every 60 residues since each participating protein has 60 residues. The discontinuous residues from the head of one fibril and the tail of the adjacent fibril are represented by the exceptionally high B′ value. The residues Lys204 and Arg220 (the 35th and 51st residues from Ser170) have higher values of B′ within a fibril due to their high flexibility (Figure 2H). The prion protein contains one intramolecular disulfide link between Cys179 and Cys214 (the 10th residue and 45th residue from Ser170). The B′ value can only be below −0.5 because of this disulfide bond. The identical pattern of B′ is seen in three simulated MD structures from implicit solvent and one simulated MD structure from explicit solvent, demonstrating similar flexibility. The Ramachandra plot of six simulated MD structures are all rich in β-strand (Supplementary Figure S1). According to the results of the implicit solvent model, fPrP_170–229_ conserves the β-strand rich structure and shares the same vibration modes as the cryo-EM structure.

**FIGURE 3:**
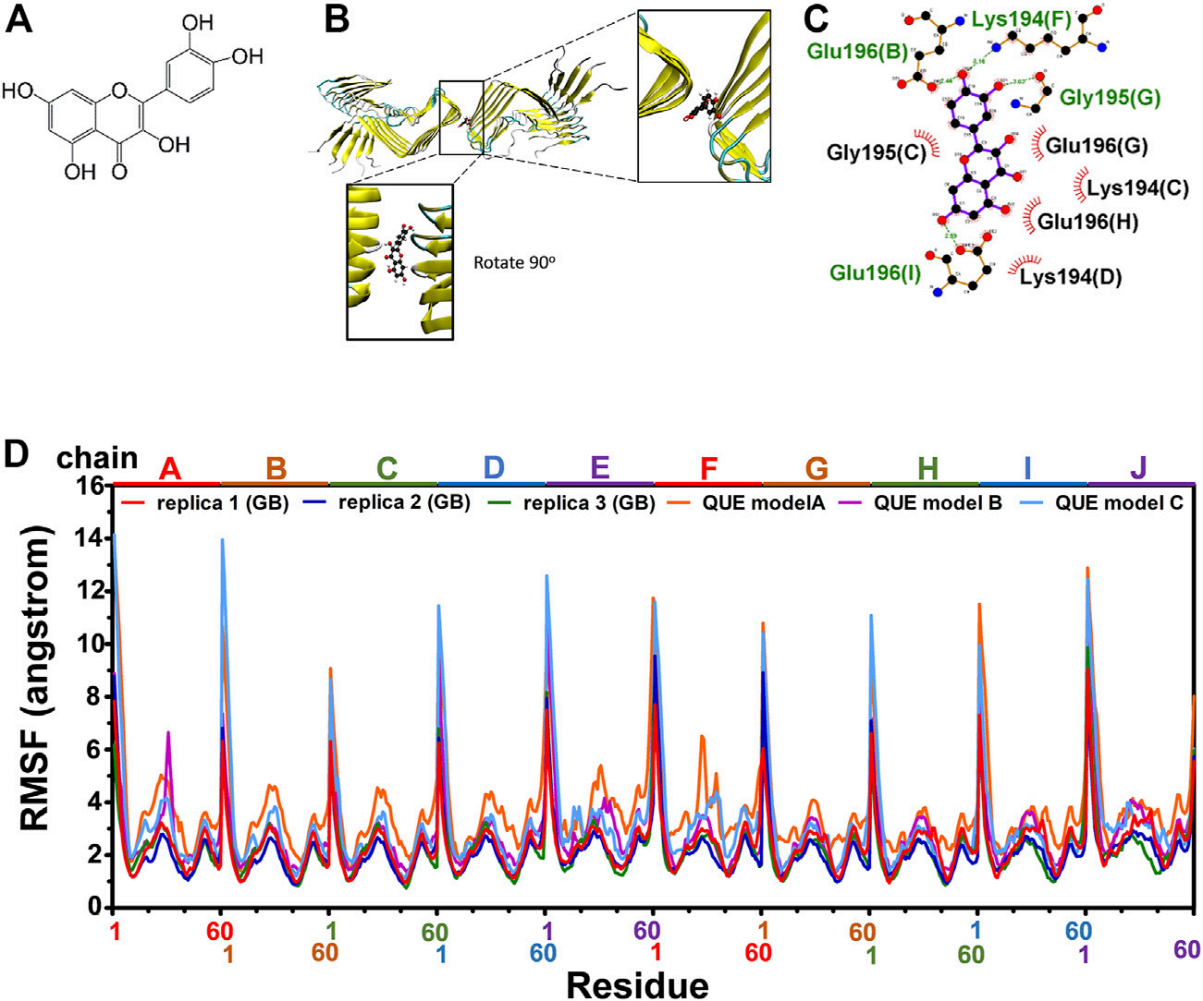
Modeling of quercetin-fPrP_170–229_ complex. **(A)** The chemical structure of quercetin. **(B)** The structure of quercetin-fPrP_170–229_ complex obtained from docking and MD. The binding cavity is shown in the enlarged frame. **(C)** Interaction network between quercetin and proximal residues. The alphabets inside of the parentheses represent fibril chains in which the corresponding residue’s location. The backbone of quercetin is shown in purple. The residues playing the role of hydrogen bond donor are labeled in green. The residues form non-bonded contact with quercetin are labeled in black with red spoked arcs. **(D)** RMSF of quercetin-fPrP_170-229_ models (QUE models A, B, and C) and RMSF of fPrP_170–229_ replicas 1, 2, and 3.

As reported previously, quercetin disaggregated mature prion fibrils (Yu and Lee, 2020). We examined the stable binding of quercetin (Figure 3A) to fPrP_170–229_ by a 162 ns MD simulation of quercetin-fPrP_170–229_ complexes. As shown in Figures 3B, C, quercetin binds to fPrP_170–229_ as a bridge between dihedral symmetric (D2) fibrils by hydrogen bonding with residues Glu196 in chain B, Gly195 in chain G, Lys194 in chain F and Glu 196 in chain I, located at the turnover of β sheets to turns. Quercetin also forms the non-bonded contact with fPrP_170–229_ at Lys194 and Gly195 in chain C, Lys194 in chain D, Glu196 in chain G and Glu196 in chain H. As shown in Figures 3B, C, eight of ten fPrP_170–229_ chains interact with quercetin, resulting in a large interaction surface. In the comparison of quercetin-fPrP_170–229_ (models A, B, and C) and fPrP_170–229_ (replicas 1, 2, and 3) in RMSF (Figure 3D), the binding of quercetin increases the flexibility of fPrP_170–229_ in the neighboring contact surface Lys194-Glu196 (the 25th to 27th residues from Ser170). Lys194 and Glu196 are critical residues for the salt bridge (Wang et al., 2020). They interact with quercetin, as shown in Figure 3C. Quercetin binding causes the rearrangement of fPrP_170–229_ because of the intermolecular repulsion. This repulsion lengthens the salt bridge and, therefore, weakens fibril stability.

### 3.2 Pharmaceutical activity of COMPs

**FIGURE 4:**
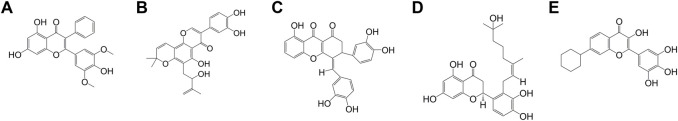
The chemical structure of COMPs **(A)** COMP **1**, **(B)** COMP **2**, **(C)** COMP **3**, **(D)** COMP **4**, and **(E)** COMP **5**.

The number of considered quercetin-like compounds (COMP) was reduced to 15 and these COMPs are eligible for Lipinski’s rule of five (Supplementary Table S2). In Supplementary Table S2, 15 COMPs have similar properties to quercetin except for logP. As judged from their high logP values, all COMPs are more lipophilic than quercetin. Table 1 is a summary of the COMPs, including topological polar surface area (TPSA, lower value represents smaller polar surface exposure), protease inhibitory potential (more negative value represents lower inhibitory activity for protease), LD_50_, hepatoxicity and docking score. The COMPs have shown high topological polar surface area (TPSA), low protease inhibitory potential, high LD_50_ and inactive of hepatotoxicity (Table 1). In addition, the pharmaceutical activity of quercetin is also listed as a reference. In TPSA, only COMPs **3** and **10** are higher than quercetin. In hepatoxicity, the COMPs **2** and **3** are less toxic than quercetin. In terms of protease inhibitory potential, COMP **3** is better than quercetin. The docking score and LD50 of 15 COMPs are better than quercetin. The low protease inhibitory potential of these COMPs **1** to **5** (Figure 4) may dispel the doubts that COMP will inhibit protease digestion, enhancing PK resistance in prion disease.

**TABLE 1:** A list of pharmacokinetics, toxicity prediction and docking score of COMPs.

COMP	TPSA (Å^2^)	Protease inhibitory potential	LD50 (mg/kg)	Hepatotoxicity	Docking score (kcal/mol)
**1**	109.36	−0.19	4000	0.71 (inactive)	−13.6
**2**	120.36	−0.18	3850	0.65 (inactive)	−12.7
**3**	148.43	−0.27	4000	0.62 (inactive)	−12.7
**4**	127.45	0.07	2000	0.80 (inactive)	−12.6
**5**	111.13	−0.16	5000	0.73 (inactive)	−12.6
**6**	120.36	−0.12	5000	0.71 (inactive)	−12.5
**7**	90.90	−0.22	2430	0.69 (inactive)	−12.4
**8**	116.45	0.06	2000	0.77 (inactive)	−12.3
**9**	111.13	−0.07	2905	0.70 (inactive)	−12.2
**10**	162.88	0.33	4000	0.69 (inactive)	−12.1
**11**	89.13	−0.20	5000	0.73 (inactive)	−12.0
**12**	128.20	−0.23	1480	0.71 (inactive)	−12.0
**13**	120.36	0.04	2500	0.69 (inactive)	−11.9
**14**	127.45	0.03	2000	0.78 (inactive)	−11.8
**15**	127.45	0.04	2000	0.80 (inactive)	−11.8
quercetin	131.36	−0.25	159	0.69 (inactive)	−9.8

The fragment based TPSA of a chemical is applied for prediction of the CNS permeation. Based on analysis of current CNS drugs, the TPSA criteria for CNS administration is lower than 60–70 Å^2^ (Kelder et al., 1999). The TPSA of quercetin is 131.36 Å^2^, higher than the criteria for CNS administration. However, it has been found that quercetin crosses blood-brain barrier (BBB) in a rat model. Quercetin transportation in the brain can be enhanced with α-tocopherol supplementation (Ferri et al., 2015). The transcellular lipophilic pathway is used by the majority of CNS drugs to cross the BBB (Abbott et al., 2006). The logP in Lipinski’s rule of five of COMPs is higher than quercetin, indicating the strong lipophilic property to support penetration across BBB. The Lipinski’s rule of five is specific to orally administrated drugs, indicating the COMPs can be administrated by oral intake to bind the PrP fibril in the PNS and tissues.

### 3.3 Redocking the solvated COMPs

As listed in Table 1, the docking score is ranged from −11.8 to −13.6 kcal/mol, more negative than the score of quercetin (−9.8 kcal/mol), indicating stronger binding in COMP-fPrP_170–229_. The docking site of COMPs **1**, **2**, **3**, **4**, and **5** with the lowest docking score are mainly located at the turnover of the β-sheets to turns as the binding of quercetin (Supplementary Figure S2). COMPs **1** model A, B, and C (abbreviated as **1**A–C), **3**B, and **3**C interact with one strand of fPrP_170–229_ unilaterally, whereas COMPs **2**A–C, **3**A, **4**A–C, and **5**A–C interact with two strands of fPrP_170–229_. Each strand is composed of fPrP_170–229_ pentamers.

### 3.4 MD simulation of COMPs-fPrP_170–229_ complexes

The three binding sites with the highest docking score of COMPs **1** to **5** were selected for MD simulation to find out their stable binding sites with fPrP_170–229_. As a result, the most common interaction is hydrogen bonding with Lys194 and Glu196 in all COMPs, except COMP **1** (Supplementary Figure S3; Table 2). This binding network is similar to that of quercetin. For COMP **1**, a similar binding is formed at Thr191, the neighboring residues of Lys194 and Glu196 in sequence. The hydrogen bonds are classified into strong (s), moderate (m), and weak (w) interactions based on their length (L_H_): strong hydrogen bond (2.2 Å ≤ L_H_ < 2.5 Å), moderate hydrogen bond (2.5 Å ≤ L_H_ < 3.2 Å), and weak hydrogen bond (3.2 Å ≤ L_H_ < 4.0 Å) interactions (Jeffrey, 1997). As a result, COMPs **1**C, **2**C, **4**C, and **5**C have weak hydrogen bonds interacting with fPrP_170–229,_ and COMPs **2**B, **3**B–C, **4**A, **4**C, and **5**A have strong hydrogen bonds interacting with fPrP_170–229_.

**TABLE 2:** The list of COMPs with their interacting fPrP_170–229_ residues and the corresponding binding energy. Each COMP was simulated in three models. Binding energy (kcal/mol).

COMP	Model	#H bond	Interacting residues [chain, hydrogen bond length (Å) (s, m or w)^a^]	Binding energy (kcal/mol)
**1**	A	3	Val203 (B, 2.67 (m)), Thr191 (C, 2.58 (m)), Thr191 (D, 3.09 (m))	−37.98 ± 2.54
	B	2	Thr191 (I, 3.15 (m)), Thr192 (J, 3.07 (m))	−28.03 ± 2.13
	C	2	Thr191 (B, 3.30 (w)), Thr193 (B, 3.32 (w))	−44.11 ± 3.14
**2**	A	2	Glu196 (G, 2.53 (m)), Glu196 (G, 2.53 (m))	−53.39 ± 3.40
	B	3	Lys194 (C, 2.91 (m)), Glu196 (F, 2.44 (s)), Glu196 (F, 2.53 (m))	−65.19 ± 3.35
	C	8	Lys194 (C, 3.04 (m)), Lys194 (C, 3.05 (m)), Lys194 (D, 3.02 (m)), Glu196 (G, 2.59 (m)), Lys194 (H, 3.23 (w)), Gly195 (I, 3.29 (w)), Glu196 (I, 2.59 (m)), Glu196 (I, 2.62 (m))	−56.07 ± 6.30
**3**	A	4	Glu196 (J, 2.51 (m)), Glu196 (J, 2.53 (m)), Glu196 (J, 2.54 (m)), Glu196 (J, 2.58 (m))	−67.19 ± 4.66
	B	4	Glu219 (B, 2.47 (s)), Arg220 (B, 3.20 (m)), Glu219 (C, 2.48 (s)), Glu219 (C, 2.50 (s))	−31.90 ± 2.49
	C	6	Asp178 (F, 2.49 (s)), Asp178 (F, 2.74 (m)), Val180 (F, 2.53 (m)), Val180 (F, 2.54 (m)), Asp178 (H, 2.60 (m)), Asp178 (H, 3.07 (m))	−18.83 ± 5.27
**4**	A	4	Lys194 (C, 2.95 (m)), Glu196 (C, 2.40 (s)), Glu196 (C, 2.52 (m)), Glu196 (F, 2.81 (m))	−56.89 ± 3.76
	B	4	Glu196 (B, 2.53 (m)), Glu196 (B, 2.64 (m)), Glu196 (D, 2.53 (m)), Glu196 (G, 3.28 (w))	−45.75 ± 6.20
	C	4	Glu196 (C, 2.48 (s)), Glu196 (C, 2.54 (m)), Glu196 (C, 3.03 (m)), Glu196 (E, 2.69 (m))	−59.53 ± 5.96
**5**	A	3	Glu196 (B, 2.76 (m)), Glu196 (F, 2.49 (s)), Glu196 (F, 2.63 (m))	−52.42 ± 3.54
	B	4	Glu196 (C, 2.53 (m)), Glu196 (C, 2.55 (m)), Lys194 (D, 3.17 (m)), Glu196 (H, 2.65 (m))	−59.39 ± 3.96
	C	5	Glu196 (B, 2.51 (m)), Glu196 (B, 2.7 (m)), Lys194 (D, 2.70 (m)), Lys194 (G, 3.31 (w)), Glu 196 (H, 2.61 (m))	−48.81 ± 6.54
quercetin		4	Glu196 (B, 2.46 (m)), Lys194 (F, 3.16 (m)), Gly195 (G, 3.03 (m)), Glu196 (I, 2.59 (m))	−39.19 ± 2.92

^a^ The strength of hydrogen bonds are classified as strong, moderate or weak abbreviating as s, m or w, respectively.

**FIGURE 5:**
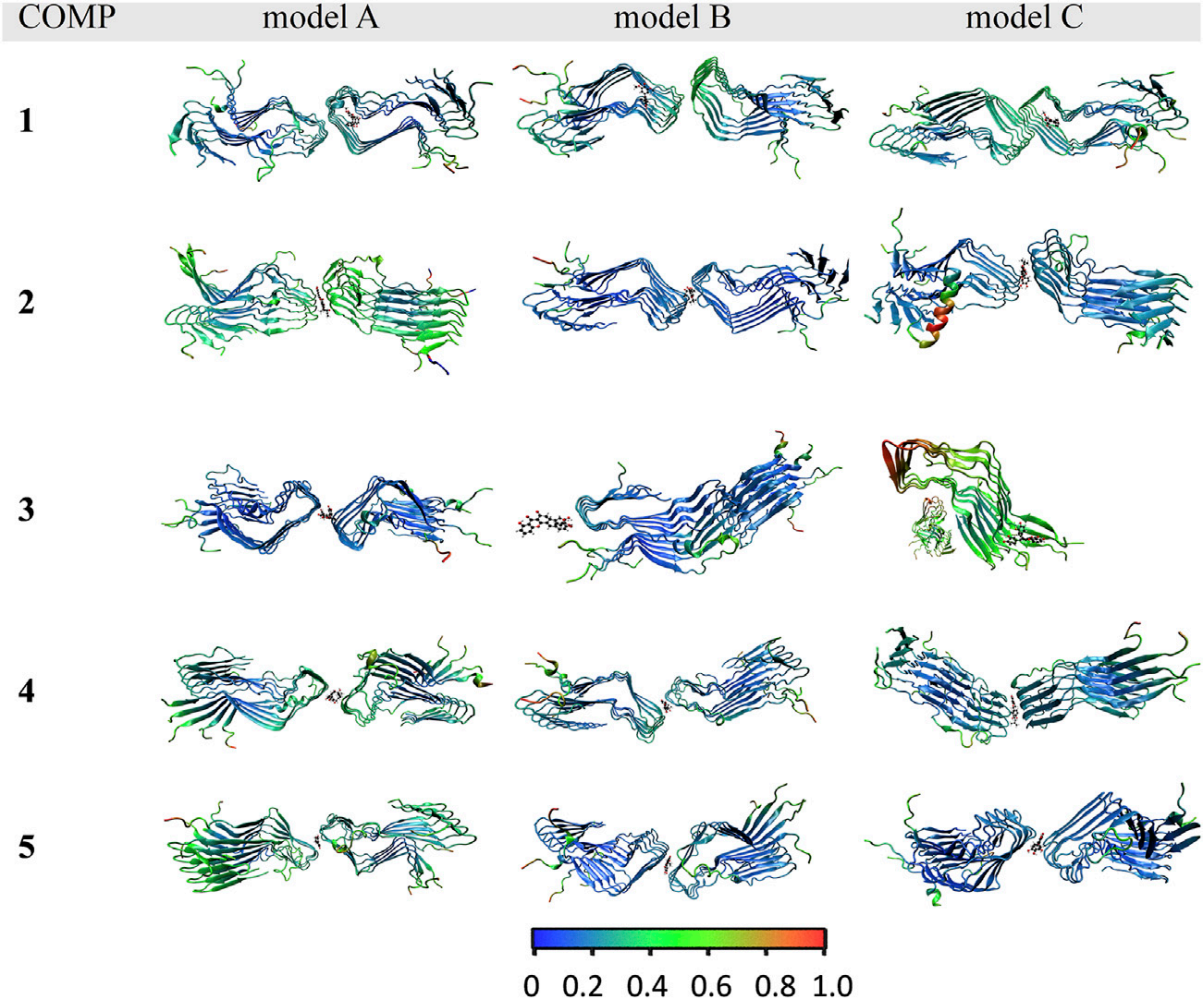
RMSF of COMP-fPrP_170–229_ mapped onto the ribbon structure of fPrP_170–229_ decamers.

**FIGURE 6:**
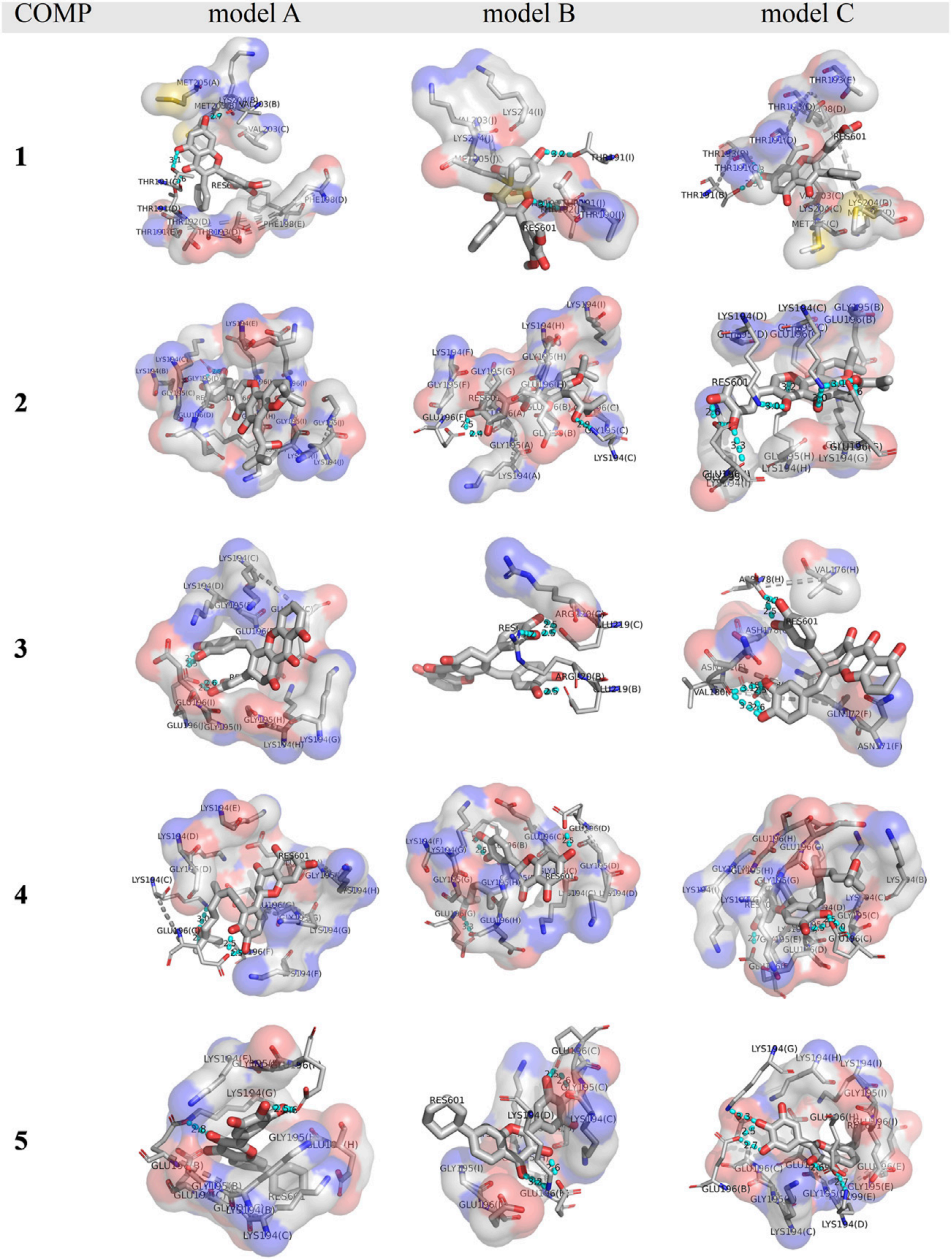
A close view of the binding cavities in COMPs **1-5** with fPrP_170–229_. The hydrogen bond between COMPs and fPrP_170–229_ is shown as a cyan dotted line. A gray dotted line connects the amino acid in the same strands of fPrP_170–229_ that acts as a hydrogen bond donor or acceptor. RES601 is the COMP in each model.

The binding affinity of various COMPs with fPrP_170–229_ was compared by their binding energy estimated using MM/GBSA (Table 2). The binding energy of COMPs **1** and **3** shows higher divergence than other compounds because of including different binding sites from docking structure (Supplementary Figure S6). In COMP **1**, the binding sites are located at the inner space or the edge of one fibril segment (Supplementary Figure S4). When COMP **3** interacts with the hinge of fPrP_170–229_ bilaterally, the binding energy is much more negative than the unilaterial ones (Supplementary Figure S4). Approximately, the binding energy at −45 kcal/mol can be a threshold for unilateral (>−45 kcal/mol) and bilateral bindings (<−45 kcal/mol) (Table 2; Supplementary Figure S4, S6). The major fluctuation is located in residues 189–202 (20th to 33rd residue from Ser170), adjacent to the compound binding site (Figure 5; Supplementary Figure S7). For the COMPs **2**, **4**, and **5**, their bindings perturbate the distal region affecting the alignment of fPrP_170–229_ or loosening the secondary structure of fPrP_170–229_ at the edge (Figure 5). In contrast, COMP **1** has very little structural alteration. In the binding energy estimation, COMP **3** shows more fluctuation and weaker binding energy compared to other compounds (Supplementary Figure S5). Upon binding to COMP **3**, the fPrP_170–229_ becomes a twisted tertiary structure or dissociated fibrils (Figure 5). In the close view of binding cavity, the two twisting ortho-phenol groups of COMPs **3**A and **3**C are inserted into fPrP_170–229_ (Figure 6). However, in COMP **3**B, the twisting ortho-phenol group interacts with fPrP_170–229_ surface (Figure 6; Supplementary Figure S3). In the COMPs **1**A-C and **3**A-C, twisting the adjacent phenol groups to form π-π stacking in some models decreases the binding energy. The electron density of π−π stacking in the aromatic plane of COMPs **1**A-C and **3**A-C may contribute to the interaction with fPrP_170–229._ COMP **1** and COMP **3** are bound at different locations from COMPs **2**, **4**, and **5** mainly due to the presence of two ortho-aromatic rings occupying more space than other COMPs. COMP **1** tends to form a pyramidal structure to bind with single side of the fibril decamers. However, two ortho-phenols of COMP **3** has to align well with face-to-face π−π interaction to fit in Lys194-Glu196 cavity. In this situation, COMP **3** maintains a solid geometry and consequently lose the degree of freedom of rotation. Otherwise, COMP **3** will fall out of the cavity and preserve large degree of freedom of rotation. Therefore, this steric restriction of COMP **3** is critical for the binding location.

The enthalpy of COMP-fPrP_170–229_ is contributed by van der Waals force and electrostatic interaction (Eq. 3; Table 3). In COMPs **1**A-C, **2**A-C, **4**B, **4**C, **5**B, and **5**C, van der Waals force is the major contributor for enthalpy. In COMPs **3**A-C, **4**A, **5**A and quercetin, the electrostatic interaction is stronger than the van der Waals force. To estimate the electrostatic interaction contributed by hydrogen bond, we calculate the average electrostatic interaction ( $E_{EEL}^{0}$ /#H bond). Correlated with the strength of the hydrogen bond in Table 2, COMPs **1**C, **2**C, and **5**C with weak hydrogen bonds are also shown weak electrostatic interaction. Similarly, COMPs **3**B and **5**A have strong hydrogen bonds and have strong electrostatic interaction ( $E_{EEL}^{0}$ /#H bond > −20 kcal/mol). The hydrogen bonds of COMPs **2**A and **3**A are all classified as moderate interaction with fPrP_170−229_, but they are at the border of the moderate and strong hydrogen bonding as judged from the hydrogen bond length.

**TABLE 3:** A list of van der Walls force (*vdW*) and electrostatic interaction (*EEL*) of enthalpy of COMP-fPrP_170–229_ complexes.

COMP	Model	$E_{vdW}^{0}$ (kcal/mol)	Non-bonded contact	$E_{EEL}^{0}$ (kcal/mol)	$E_{EEL}^{0}$ /#H bond (kcal/mol)
**1**	A	−40.77 ± 3.03	9	-32.66 ± 4.63	−10.88
	B	−32.77 ± 2.42	7	-6.52 ± 2.86	−3.25
	C	−51.31 ± 3.09	10	-19.32 ± 7.39	−9.66
**2**	A	−52.35 ± 3.79	14	-40.18 ± 5.59	−20.09
	B	−66.87 ± 3.69	14	-50.65 ± 4.95	−16.88
	C	−56.78 ± 3.60	9	-56.04 ± 14.50	−7.01
**3**	A	−55.18 ± 3.65	10	-89.33 ± 10.83	−22.33
	B	−2.04 ± 3.64	1	-88.43 ± 4.95	−22.11
	C	−18.42 ± 6.06	5	-31.58 ± 15.94	−5.26
**4**	A	−56.03 ± 3.21	10	-64.62 ± 6.66	−16.16
	B	−50.78 ± 3.46	11	-36.45 ± 11.96	−9.11
	C	−58.69 ± 5.18	15	-52.39 ± 15.27	−13.10
**5**	A	−46.25 ± 3.83	10	-61.15 ± 7.18	−20.38
	B	−50.38 ± 3.72	7	-48.87 ± 6.57	−12.22
	C	−55.04 ± 3.48	12	-27.56 ± 9.57	−5.51
quercetin		−32.44 ± 3.36	5	-66.07 ± 6.52	−16.52

**FIGURE 7:**
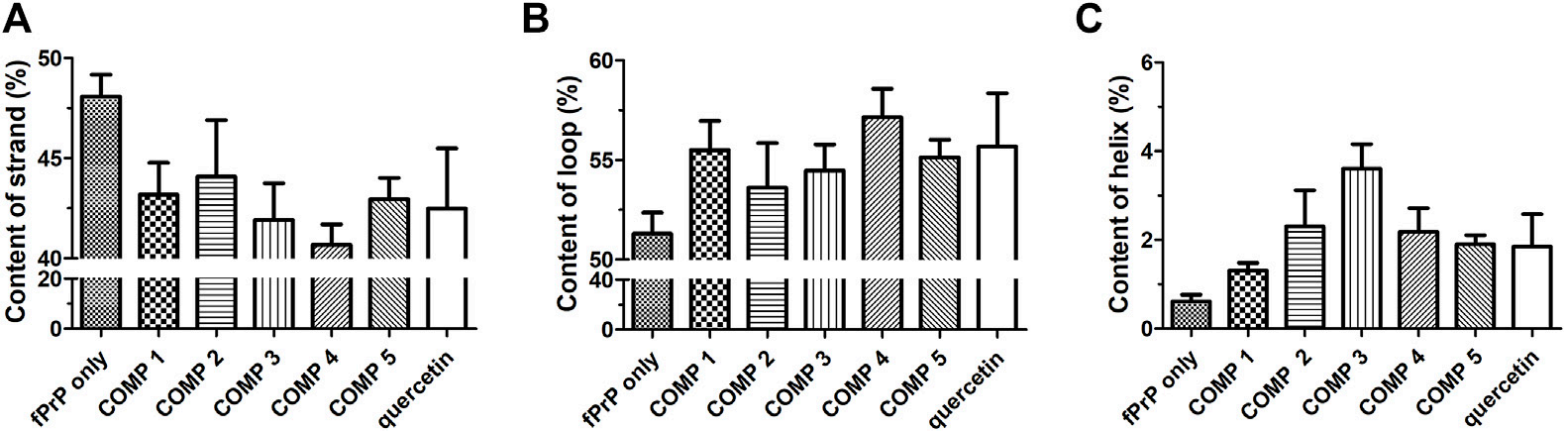
DSSP analysis of the secondary structure contents of **(A)** strand, **(B)** loop, and **(C)** helix in ligand-fPrP_170–229_ (*n* = 3).

The stability of the PK-resistant amyloid core is affected by the structural content (Bocharova et al., 2006; Vázquez-Fernández et al., 2012). The structures of COMP-fPrP_170−229_ complexes were analyzed by DSSP as eight types of conformation (Supplementary Figure S8). In COMPs **1**C, **2**C, **3**B–C, **4**B, the COMP-fPrP_170−229_ complexes are still rich in α-helices. These eight types of conformational features were simplified into three categories: helix (alpha-helix, 3_10_ helix and π-helix), β-strand (parallel and anti-parallel β-sheets), and loop (none structure, turn and bend) (Figure 7). The content of the β-strand in fPrP_170−229_ is significantly decreased after the binding of COMPs. (Figure 7A). The binding of COMPs increases loop and helix contents in fPrP_170−229_ (Figures 7B, C). Quercetin is shown to be highly effective for prion fibril treatment (Yu and Lee, 2020). Herein, we found that quercetin binding decreases strand content and increases loop and helix contents in fPrP_170−229_. As aforementioned, the structural content affects fibril’s PK sensitivity. Fibrils with unstable structures are not capable to serve as templates for following fibrillation. Comparing to strands and helices, loops are more exposued to proteinase digestion in prion fibrils (Vázquez-Fernández et al., 2012). Combining the results of binding energy and structural contents, COMPs are more effective to alternate fibril structure resulting loss of template function in following fibrillation than quercetin.

## 4 Discussion

The structure of mammalian prion fibril is extremely important for structure-based drug design. In accordance with the source of cryo-EM structures, fPrP_170−229_ and fPrP_106−145_ were converted from two different *in vitro* system, while fPrP_95−227_ was converted from an *in vivo* system. Wang and colleagues found that fPrP_170−229_ is structurally stabilized by a salt bridge formed by opposing subunits, resulting in the formation of hydrophobic cavity between contact surfaces (Wang et al., 2020). Different from Wang’s stacking model of fPrP_170−229_ in neutral pH, Glynn and coworkers found that fPrP_106−145_ is stacked in parallel and attached neighboring fibrils with the N-terminal Thr107-Leu125 (Glynn et al., 2020). After the cryo-EM results of fPrP_170−229_, Requena and coworkers rebuilt the 4RβS model of fully glycosylated human PrP amyloid (Spagnolli et al., 2020). Through the tail end of C-terminal PrP^Sc^ in 4RβS model, PrP^Sc^ successfully induces structural conversion of PrP^C^ into PrP^Sc^ in MD simulation. To check the reliability of fPrP_170−229_ from an *in vitro* system, we compared the fPrP_170−229_ structure with the fPrP_95−227_ structure from an *in vivo* 263K mouse model. The fPrP_170−229_ has the similar secondary structure and tertiary structure in the comparison with fPrP_95−227_ (PDB 7LNA, Supplementary Figure S9) (Kraus et al., 2021). As with fPrP_95−227_, fPrP_170−229_ exposes two glycosylation sites (Asn181 and Asn197) on the water-accessible surface. The E196K point mutation of PrP fibril (fPrP_175-217_, PDB 7DWV, Supplementary Figure S10), was shown to have a large alteration in dimerized region (Wang et al., 2021). Two positive charge residues, K194 and K196, decrease the fibril stability by disrupting salt bridge directly and rearranging the local environment of interaction network (Wang et al., 2021). This evidence indicates the importance of the salt bridge of K194 and E196 for building up the fibril stability.

According to a previous study based on antibodies against different PrP regions, the flexible N-terminal tail mediates toxicity and globular C-terminal domain induces toxicity (Sonati et al., 2013). In the inherited prion diseases, the residues of point mutation are mainly located in the C-terminus (Prusiner, 1998). The infectious fragment of PrP^Sc^ has been identified as a part of the PK-resistant sequence (Wang et al., 2018). In the GPI-anchor free Scrapie infected mouse model, the highly PK-resistant fragment is the region from residue 179 to the C-terminal end (Vázquez-Fernández et al., 2012). In the comparison of protein misfolding cyclic amplification (PMCA) and Gdn-HCl induced conversion through hydrogen/deuterium exchange, the products from these two different conditions indicate the same highly protected region at residues 170–213 (Smirnovas et al., 2009). According to these studies, the C-terminal PrP has the most important role in prion infection and proteolytic resistance. In this study, the cryo-EM structure includes this highly protected region. Therefore, fPrP_170–229_ is an ideal target to analyze the effect of drug candidates after their binding to the fibril core.

In MD simulation, the torsions of amino acid are recorded in the force field affecting the secondary structure formation. Currently, the bias of force fields has been found to tend to overestimate α-helix in the course of protein folding (Best et al., 2008). The MD simulation is based on the solid empirical data from experimental studies. For MD simulation of intrinsically disordered proteins (IDP) or fibrils, the force field is the most critical for the reliability of the results. Many force fields and explicit water models in MD simulation have been investigated in IDPs, such as Aβ and α-synuclein (Akbayrak et al., 2020). Akbayrak and colleagues have reviewed early-developed Amber force fields. In the use of the Amber99 force field, Aβ_16–22_ favors the formation of helical structures in monomeric, dimeric, and trimeric forms. In contrast to the experimental findings, Aβ_16–22_ cannot fold into β-sheets. Three explicit solvent models (SPC, TIP3P, and TIP4P) fold Aβ_40_ into an α-helical structure when using the Amber03 force field. These problems were corrected in Amber99SB (a revised version of Amber99 (Hornak et al., 2006) along with TIP3P, as judged from the simulation of Aβ_42_. Consistent with the experimental observation, α-synuclein forms an α-helical structure in the N-terminal region in MD simulation with Amber99*-ILDN or Amber99SB. In the MD tests of dimeric Aβ_16–22_ fibrils, five newer force fields including Amber99-ILDN, Amber14SB, CHARMM22*, CHARMM36 and CHARMM36m were chosen for the study of amyloid peptide fibril-assembly (Man et al., 2019). In the modeling with Amber14SB, it was observed that there was a low transition in the secondary structure between anti-parallel β-sheet and parallel β-sheet in fibril dimer formation (Man et al., 2019). Shortly speaking, Amber14SB is a revised version to calibrate the side chain and backbone parameters of Amber99SB (Maier et al., 2015). Based on these studies, Amber14SB is suitable for resolving protein structures.

In the comparison of cryo-EM structure, similar Β’ value shown in Figure 2 indicates the suitability of the force field in this work. Solvent is critical for molecular interactions. The reliability of implicit solvent model has been investigated. Implicit solvent model has been used for simulation of paired helical filament 6 (PHF6) fragment from tau protein (Huang and Stultz, 2007) and Aβ fibril (Gurry and Stultz, 2014). The former study indicates that implicit solvent (generalized born model) is in good agreement with TIP3P explicit solvent model in Ramachandran plot and free-energy profile. In addition, the results obtained from implicit solvent model are consistent with the experimental observation of the structural change. The later study provides the elongation of Aβ fibril induced by Aβ peptide forming β-hairpin structure and stacking with fibril predicted based on the implicit model. This prediction is consistent with the results of explicit solvent model and experimental observation.

In the pathogenesis of prion diseases, the accumulation of oligomers or fibrils induces production of ROS, resulting in an inflammatory reaction with gliosis and neuron loss (Aguzzi and Zhu, 2017). Many studies have proposed various mechanisms of prion diseases, and it is widely believed that microglia activation is the early stage of prion diseases before neuron loss and spongiform biopsy, stepwise (Aguzzi and Zhu, 2017). It was reported that PrP_106–126_ fibril activates microglia secreting pro-inflammatory cytokines (i.e.: TNF-α, IL-1β, IL-6) and upregulates NO synthase, which produces NO to induce neuronal cell death in BV2-primary microglia coculture (Kouadir et al., 2012). To decrease the neuronal cell death induced by microglia, the polyphenols are excellent in the elimination of ROS or RNS, resulting in a decrease in the cytokine secretion from microglia cells. The polyphenols from magnolia have been found to inhibit ROS and RNS production, and then to attenuate oxidative stress and inflammatory responses in neurons and microglia (Chuang et al., 2013). In PrP fibril treated Neuro-2a cells, the clearance of ROS and significantly increased cell viability were observed after quercetin treatment (Yu and Lee, 2020).

Previous studies have indicated that prion protein is stabilized by Glu196, forming intramolecular salt bridges with Arg156, His187 (Lee and Chang, 2019) or Lys194 (Sengupta et al., 2017). Based on the cryo-EM results (Wang et al., 2020), the intermolecular interaction between Lys194 and Glu196 is critical for fibril stability. The transition of Lys194 and Glu196 from intramolecular to intermolecular interaction is important to the PrP^C^-PrP^Sc^ conversion. In our MD simulation, quercetin accelerates the low toxic and PK-sensitive fibril formation by weakening the fibril structure and interacting with PrP protein at Glu196 (Yu et al., 2022). The same binding residue consists of the MD result from the quercetin-fPrP_170–229_ complex in this work. An early study has shown that 2-pyrrolidin-1-yl-N-[4-[4-(2-pyrrolidin-1-yl-acetylamino)-benzyl]-phenyl]-acetamide (GN8), a diphenylmethane derivative, interacts with Glu196, resulting in an effectively prolonged lifespan of PrP^Sc^ infected mice (Kuwata et al., 2007). Furthermore, E196K mutant induces the structural change extending the binding surface to a stable fibril structure (Wang et al., 2021). This evidence indicates that Glu196 is critical for PrP stability and drug-targeting. In this work, we have found some quercetin-like molecules interacting with the critical residues, including Lys194 and Glu196, to reduce β-strand content.

**FIGURE 8:**
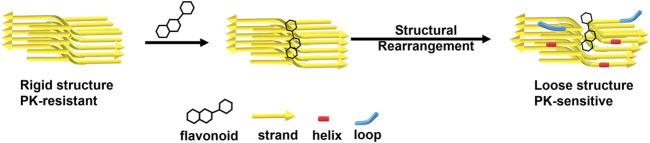
A representative scheme of flavonoids disaggregates prion fibrils by decreasing β-sheets and increasing helices and loops.

The PK-resistant fragments are located in the β-sheet region (Vázquez-Fernández et al., 2012). Our MD results indicate that some quercetin-like compounds have strong binding to the C-terminal region of fPrP_170–229_ and this binding weakens the β-strand. In the nucleation-dependent protein polymerization theory, the nuclei are critical to the ongoing conversion. The nuclear morphology is dependent on the strains of PrP^Sc^. Therefore, the structure of PrP fibrils is critical in PrP^C^-PrP^Sc^ conversion (Bessen et al., 1995). Combining the MD results from this work and critical morphology role of PrP^Sc^, we demonstrate that the change of fibril secondary structure affects the efficiency of fibril polymerization (Figure 8). Therefore, COMP-binding induced structural change is helpful in relieving the prion transmission.

## 5 Conclusion

Previously, many structure-based anti-prion drug developments were performed based on the structure of prion protein monomer. In this study, our target is prion fibrils rather than protein monomers. Thoughtfully considering the clinical potential of the searched molecules, we performed structure-based virtual screening, Lipinski’s rule of five, toxicity prediction, molecular docking and molecular dynamics to find potential anti-prion fibril flavonoids. Quercetin is well known to disaggregate many kinds of amyloid fibrils, including prion fibrils. Therefore, the effect of searched anti-prion fibril flavonoids is compared with quercetin. The selected five COMPs with pharmacological activity are eligible for Lipinski’s rule of five. Their binding energies to fPrP_170–229_ are stronger than quercetin. The major binding site of COMPs with fPrP_170–229_ is on the contacting surface of fPrP_170–229_ for dimerization at Lys194 and Glu196. Most COMPs form strong interactions with fPrP_170–229_ by van der Waals force. COMP **2**A, **3**A, **3**B and **5**A have strong electrical interactions with fPrP_170–229_ contributed by strong or moderate hydrogen bonds. COMPs **3**, **4**, and **5** induce fPrP_170–229_ to decrease the β-strand content significantly and to form helices or loops. The COMP **1** induces fPrP_170–229_ to form helices. These COMPs have great potential for further *in vitro* and *in vivo* evaluation. Overall, this work takes a big stride in the treatment for prion diseases.

## Data Availability

The original contributions presented in the study are included in the article/Supplementary Material, further inquiries can be directed to the corresponding author.
