# Supplementary material for: Combination of structure-based virtual screening, molecular docking and molecular dynamics approaches for the discovery of anti-prion fibril flavonoids
Source: Front Mol Biosci. 2023 Jan 5;9:1088733. doi: 10.3389/fmolb.2022.1088733 (PMC9849400; doi:10.3389/fmolb.2022.1088733)
Supplement: Supplementary file 1 [file DataSheet1.docx]

Supplementary Material

Table S1 A list of 100 quercetin-like molecules with high docking score.

| 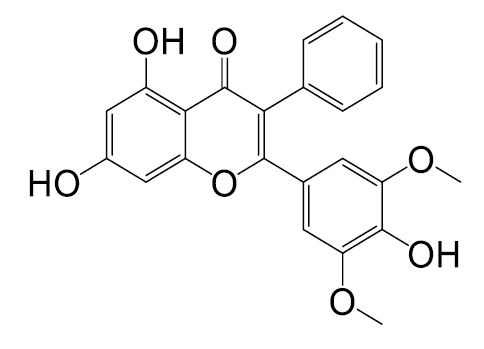  5,7-dihydroxy-2-(4-hydroxy-3,5-dimethoxyphenyl)-3-phenylchromen-4-one  C_23_H_18_O_7_  PubChem: 66779341 | 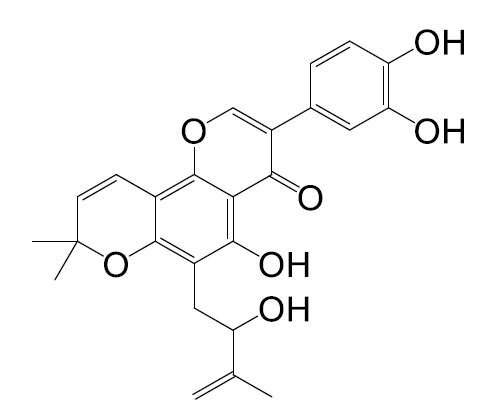  3-(3,4-dihydroxyphenyl)-5-hydroxy-6-(2-hydroxy-3-methyl-3-butenyl)-8,8-dimethyl-4H,8H-benzo[1,2-b:3,4-b']dipyran-4-one  C_25_H_24_O_7_  101676241 | 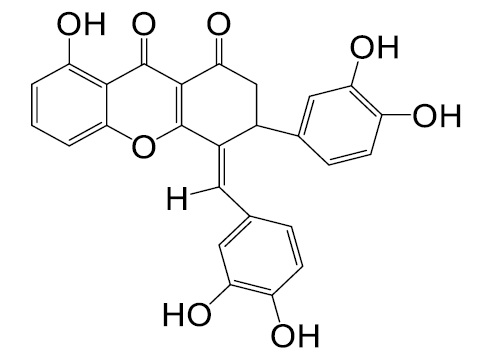  (4*E*)-3-(3,4-dihydroxyphenyl)-4-[(3,4-dihydroxyphenyl)methylidene]-8-hydroxy-2,3-dihydroxanthene-1,9-dione  C_26_H_18_O_8_  102144697 | 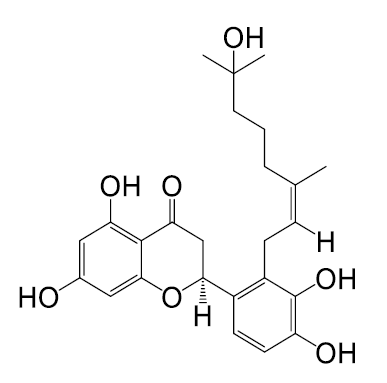  (2S)-2-[3,4-dihydroxy-2-[(Z)-7-hydroxy-3,7-dimethyloct-2-enyl]phenyl]-5,7-dihydroxy-2,3-dihydrochromen-4-one  C_25_H_30_O_7_  10411087 | 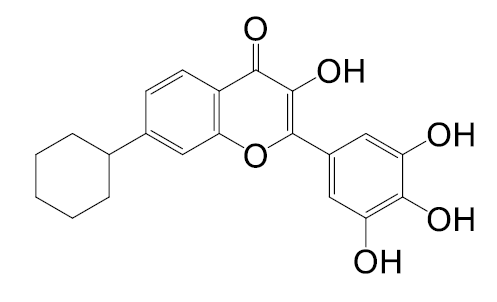  7-cyclohexyl-3-hydroxy-2-(3,4,5-trihydroxyphenyl)chromen-4-one  C_21_H_20_O_6_  56963466 | 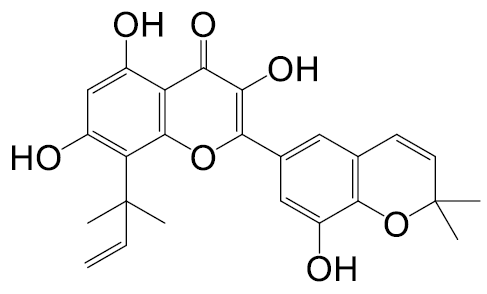  3,5,7-trihydroxy-2-(8-hydroxy-2,2-dimethylchromen-6-yl)-8-(2-methylbut-3-en-2-yl)chromen-4-one  C_25_H_24_O_7_  10342975 |
| --- | --- | --- | --- | --- | --- |
| 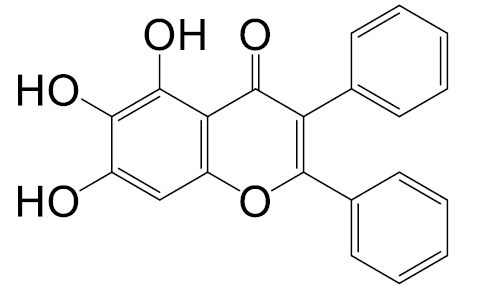  5,6,7-trihydroxy-2,3-diphenylchromen-4-one  C_21_H_14_O_5_  1024700 | 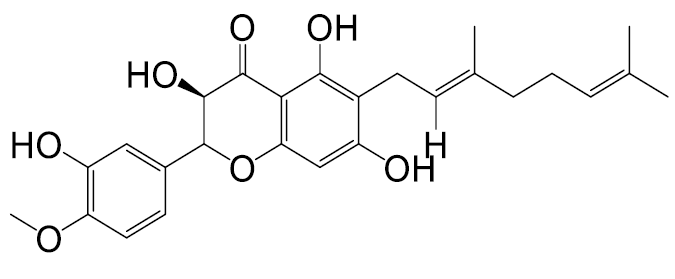  (3*R*)-6-[(2*E*)-3,7-dimethylocta-2,6-dienyl]-3,5,7-trihydroxy-2-(3-hydroxy-4-methoxyphenyl)-2,3-dihydrochromen-4-one  C_26_H_30_O_7_  122527114 | 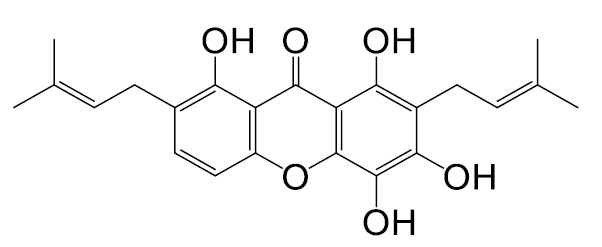  1,3,4,8-tetrahydroxy-2,7-bis(3-methylbut-2-enyl)xanthen-9-one  C_23_H_24_O_6_  118349804 | 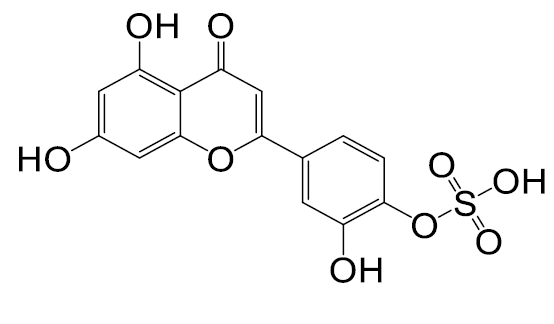  [4-(5,7-dihydroxy-4-oxochromen-2-yl)-2-hydroxyphenyl] hydrogen sulfate  C_15_H_10_O_9_S  44258152 | 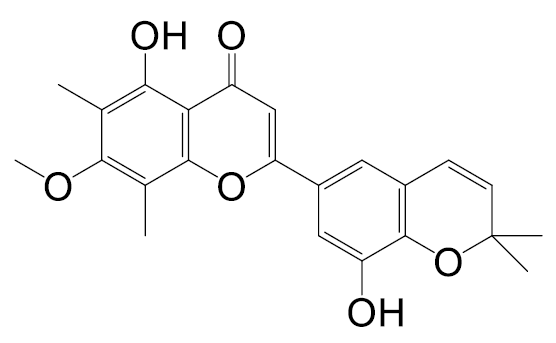  5-hydroxy-2-(8-hydroxy-2,2-dimethylchromen-6-yl)-7-methoxy-6,8-dimethylchromen-4-one  C_23_H_22_O_6_  44258380 | 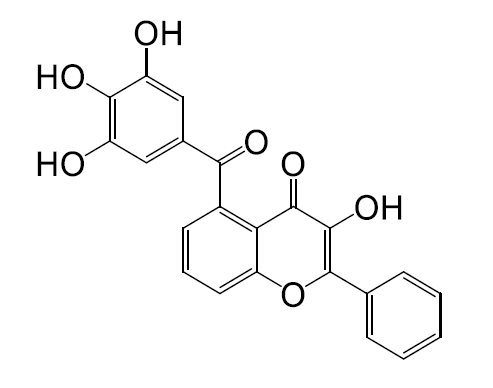  3-hydroxy-2-phenyl-5-(3,4,5-trihydroxybenzoyl)chromen-4-one  C_22_H_14_O_7_  12970394 |

Table S1 (continued) A list of 100 quercetin-like molecules with high docking score.

| 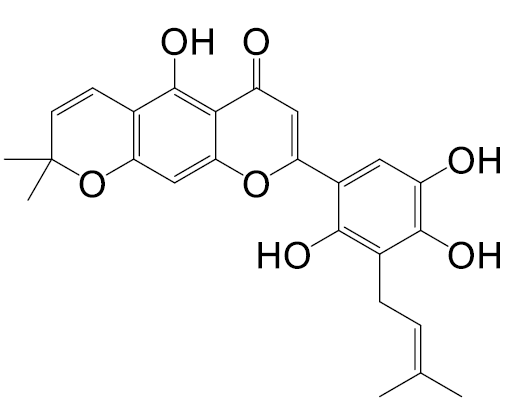  5-hydroxy-2,2-dimethyl-8-[2,4,5-trihydroxy-3-(3-methylbut-2-enyl)phenyl]pyrano[3,2-g]chromen-6-one  C_25_H_24_O_7_  14259062 | 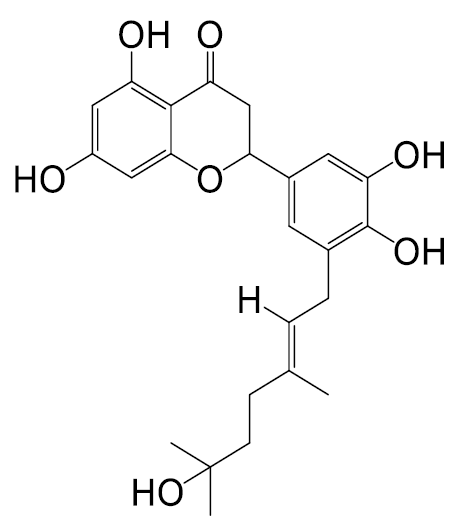  2-[3,4-dihydroxy-5-[(E)-6-hydroxy-3,6-dimethylhept-2-enyl]phenyl]-5,7-dihydroxy-2,3-dihydrochromen-4-one  C_24_H_28_O_7_  22297676 | 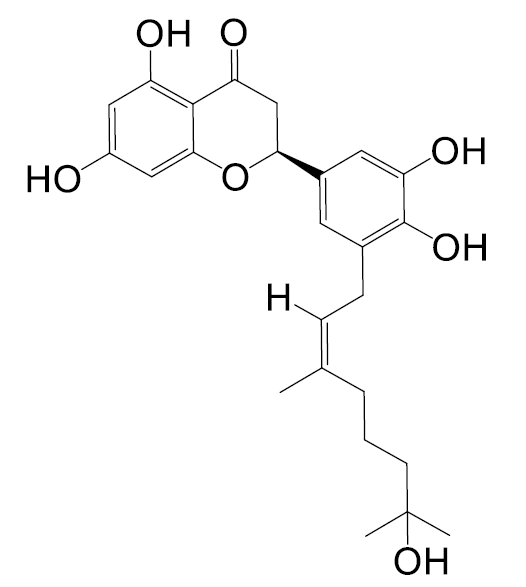  (2*S*)-2-[3,4-dihydroxy-5-[(*Z*)-7-hydroxy-3,7-dimethyloct-2-enyl]phenyl]-5,7-dihydroxy-2,3-dihydrochromen-4-one  C_25_H_30_O_7_  24751435 | 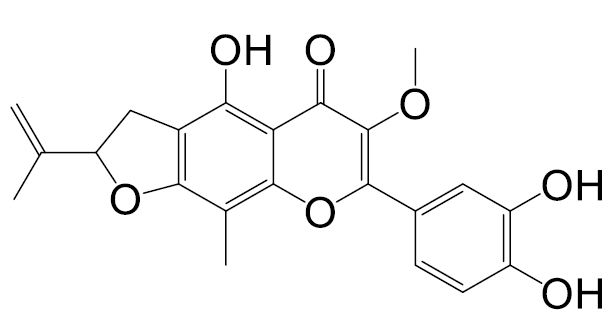  8-C-Methylvelloquercetin 3-methyl ether  C_22_H_20_O_7_  44259675 | 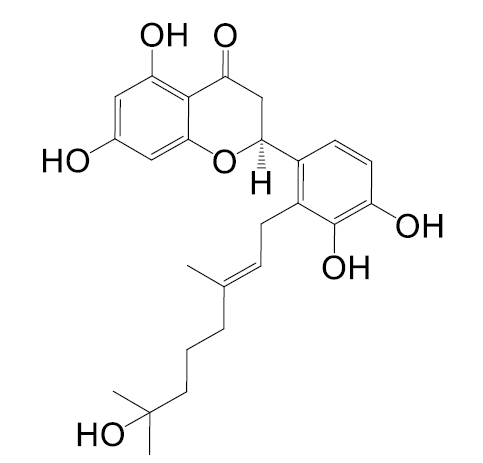  (2*S*)-2-[3,4-dihydroxy-2-(7-hydroxy-3,7-dimethyloct-2-enyl)phenyl]-5,7-dihydroxy-2,3-dihydrochromen-4-one  C_25_H_30_O_7_  90815392 | 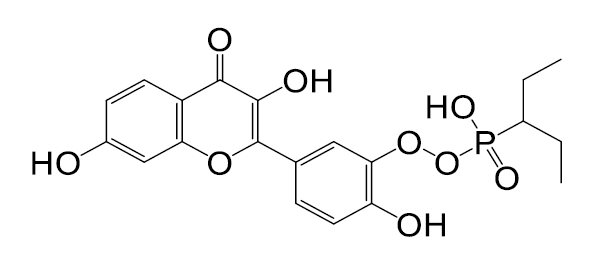  [5-(3,7-dihydroxy-4-oxochromen-2-yl)-2-hydroxyphenyl]peroxy-pentan-3-ylphosphinic acid  C_20_H_21_O_9_P  91473052 |
| --- | --- | --- | --- | --- | --- |
| 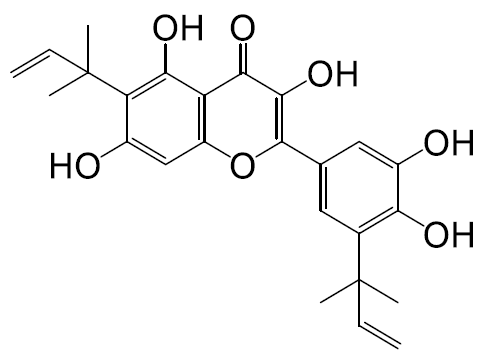  2-[3,4-dihydroxy-5-(2-methylbut-3-en-2-yl)phenyl]-3,5,7-trihydroxy-6-(2-methylbut-3-en-2-yl)chromen-4-one  C_25_H_26_O_7_  91249671 | 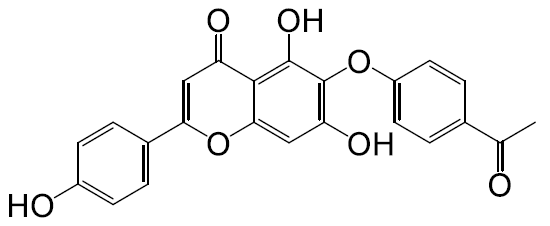  6-(4-acetylphenoxy)-5,7-dihydroxy-2-(4-hydroxyphenyl)chromen-4-one  C_23_H_16_O_7_  91427882 | 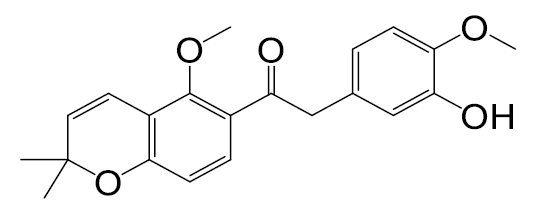  2-(3-hydroxy-4-methoxyphenyl)-1-(5-methoxy-2,2-dimethylchromen-6-yl)ethanone  C_21_H_22_O_5_  122700651 | 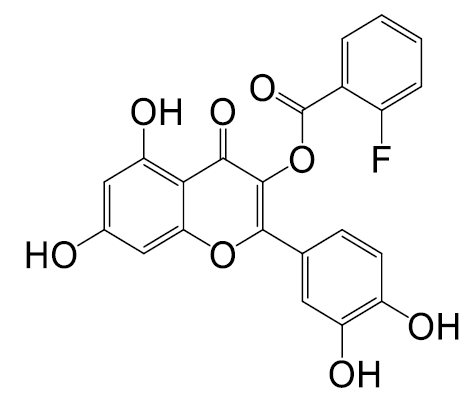  [2-(3,4-dihydroxyphenyl)-5,7-dihydroxy-4-oxochromen-3-yl] 2-fluorobenzoate  C_22_H_13_FO_8_  129904923 | 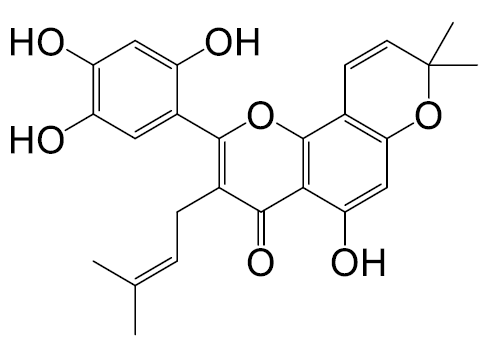  5-hydroxy-8,8-dimethyl-3-(3-methylbut-2-enyl)-2-(2,4,5-trihydroxyphenyl)pyrano[2,3-h]chromen-4-one  C_25_H_24_O_7_  5481962 | 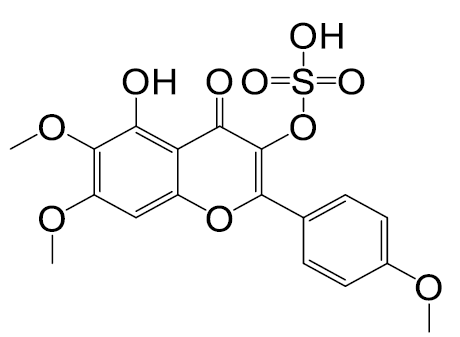  [5-hydroxy-6,7-dimethoxy-2-(4-methoxyphenyl)-4-oxochromen-3-yl] hydrogen sulfate  C_18_H_16_O_10_S  14630674 |

Table S1 (continued) A list of 100 quercetin-like molecules with high docking score.

| 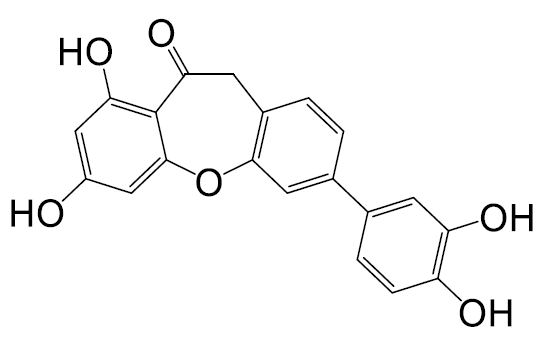  9-(3,4-dihydroxyphenyl)-2,4-dihydroxy-6*H*-benzo[b][1]benzoxepin-5-one  C_20_H_14_O_6_  20631753 | 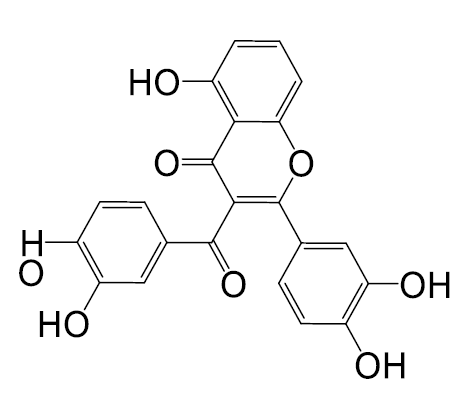  3-(3,4-dihydroxybenzoyl)-2-(3,4-dihydroxyphenyl)-5-hydroxychromen-4-one  C_22_H_14_O_8_  23631500 | 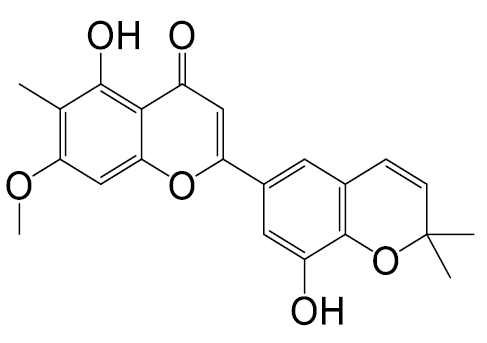  5-hydroxy-2-(8-hydroxy-2,2-dimethylchromen-6-yl)-7-methoxy-6-methylchromen-4-one  C_22_H_20_O_6_  44258379 | 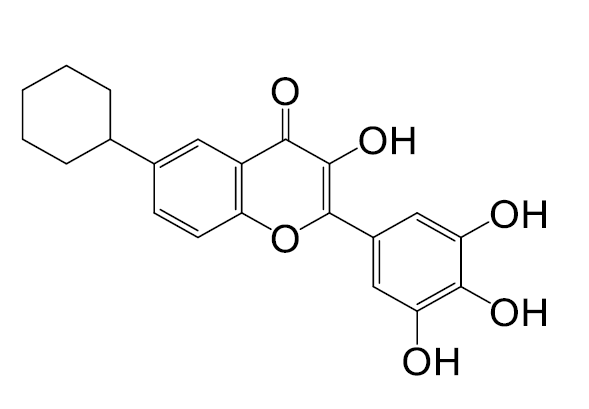  6-cyclohexyl-3-hydroxy-2-(3,4,5-trihydroxyphenyl)chromen-4-one  C_21_H_20_O_6_  56963465 | 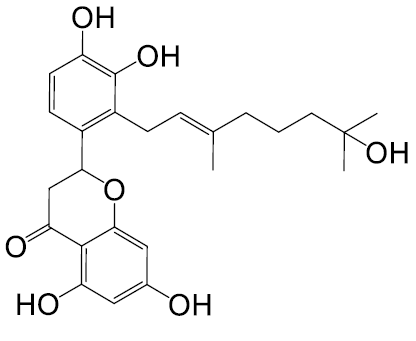  2-[3,4-dihydroxy-2-(7-hydroxy-3,7-dimethyloct-2-enyl)phenyl]-5,7-dihydroxy-2,3-dihydrochromen-4-one  C_25_H_30_O_7_  66839900 | 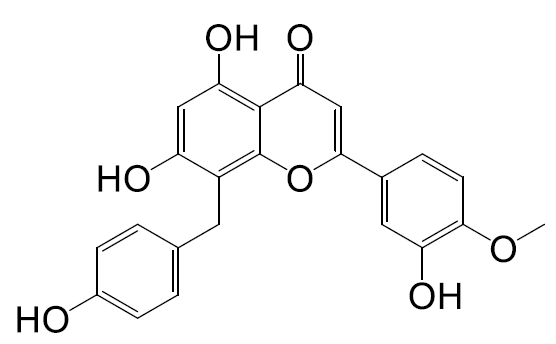  5,7-dihydroxy-2-(3-hydroxy-4-methoxyphenyl)-8-[(4-hydroxyphenyl)methyl]chromen-4-one  C_23_H_18_O_7_  101916307 |
| --- | --- | --- | --- | --- | --- |
| 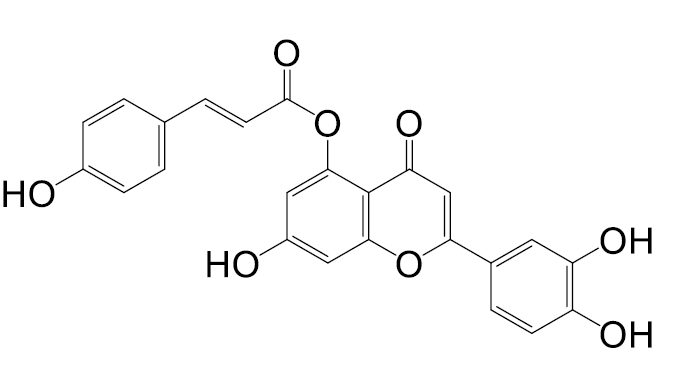  [2-(3,4-dihydroxyphenyl)-7-hydroxy-4-oxochromen-5-yl] 3-(4-hydroxyphenyl)prop-2-enoate  C_24_H_16_O_8_  123616566 | 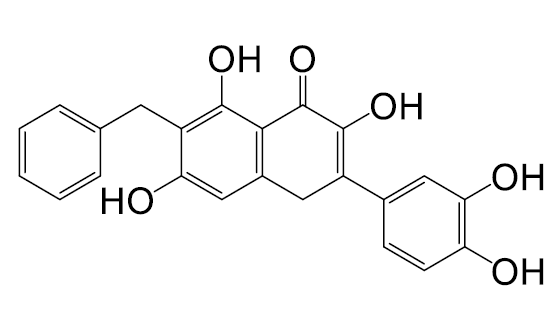  6-benzyl-2-(3,4-dihydroxyphenyl)-3,5,7-trihydroxychromen-4-one  C_22_H_16_O_7_  129760288 | 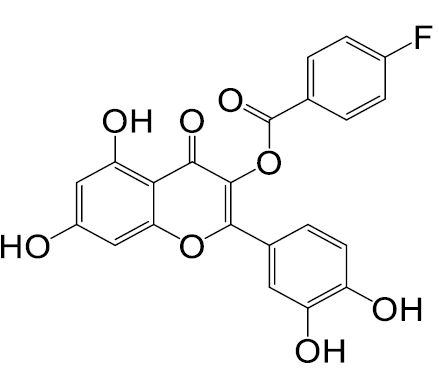  [2-(3,4-dihydroxyphenyl)-5,7-dihydroxy-4-oxochromen-3-yl] 4-fluorobenzoate  C_22_H_13_FO_8_  129904893 | 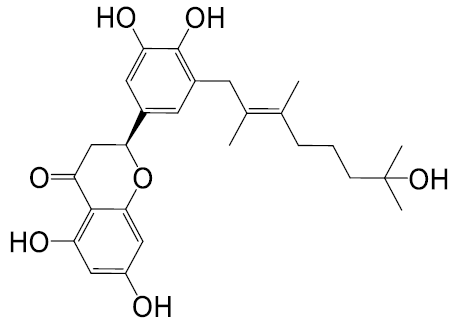  (2S)-2-[3,4-dihydroxy-5-[(E)-7-hydroxy-3,7-dimethyloct-2-enyl]phenyl]-5,7-dihydroxy-2,3-dihydrochromen-4-one  C_25_H_30_O_7_  637252 | 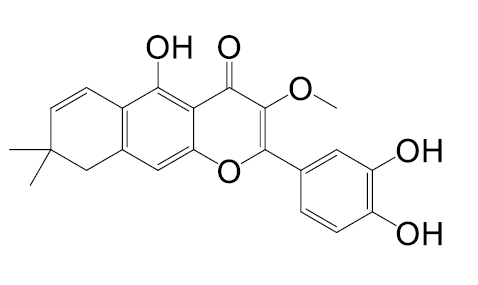  8-(3,4-dihydroxyphenyl)-5-hydroxy-7-methoxy-2,2-dimethylpyrano[3,2-g]chromen-6-one  C_21_H_18_O_7_  9977173 | 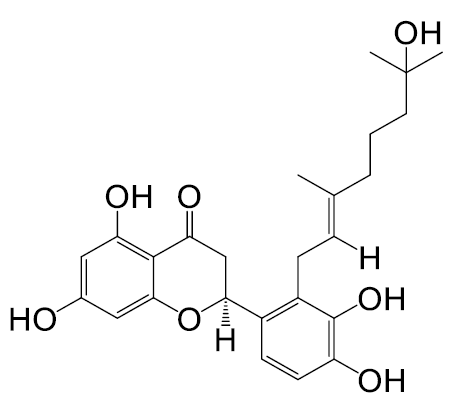  (2*S*)-2-[3,4-dihydroxy-2-[(*E*)-7-hydroxy-3,7-dimethyloct-2-enyl]phenyl]-5,7-dihydroxy-2,3-dihydrochromen-4-one  C_25_H_30_O_7_  10950300 |

Table S1 (continued) A list of 100 quercetin-like molecules with high docking score.

| 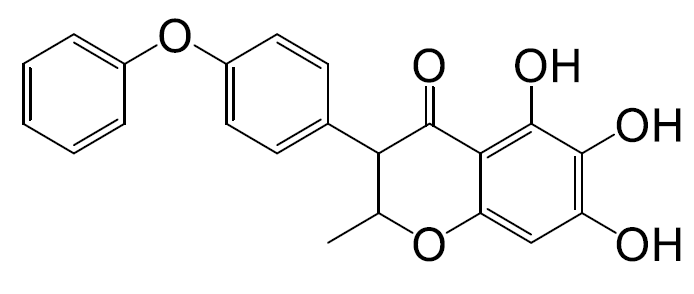  5,6,7-trihydroxy-2-methyl-3-(4-phenoxyphenyl)chromen-4-one  C_22_H_16_O_6_  58749545 | 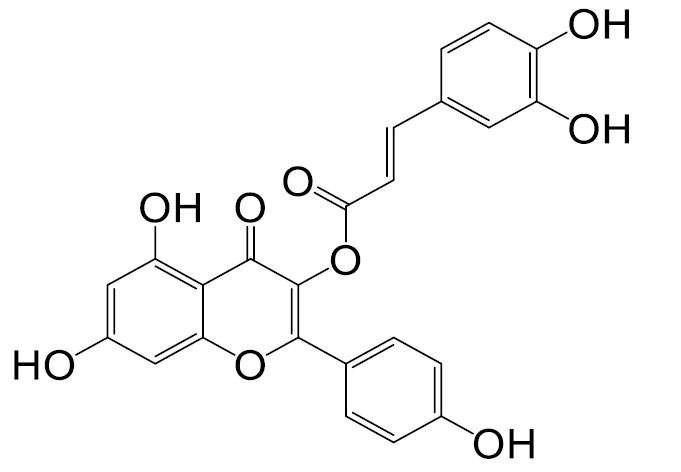  [5,7-dihydroxy-2-(4-hydroxyphenyl)-4-oxochromen-3-yl] 3-(3,4-dihydroxyphenyl)prop-2-enoate  C_24_H_16_O_9_  69307307 | 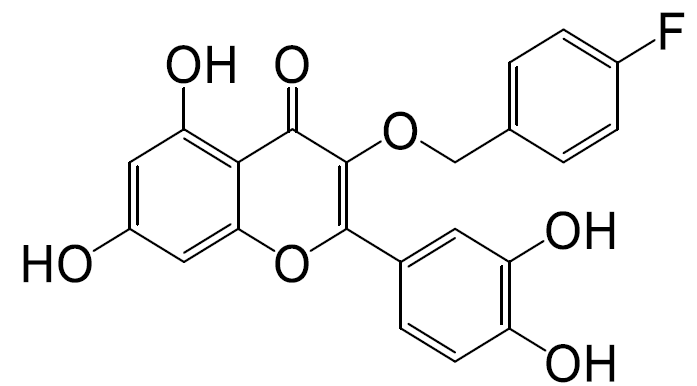  2-(3,4-dihydroxyphenyl)-3-[(4-fluorophenyl)methoxy]-5,7-dihydroxychromen-4-one  C_22_H_15_FO_7_  90643984 | 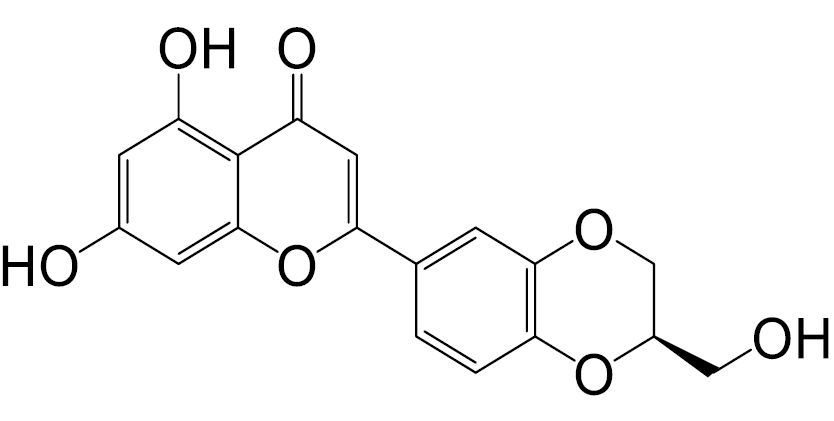  5,7-dihydroxy-2-[(2R)-2-(hydroxymethyl)-2,3-dihydro-1,4-benzodioxin-6-yl]chromen-4-one  C_18_H_14_O_7_  101064811 | 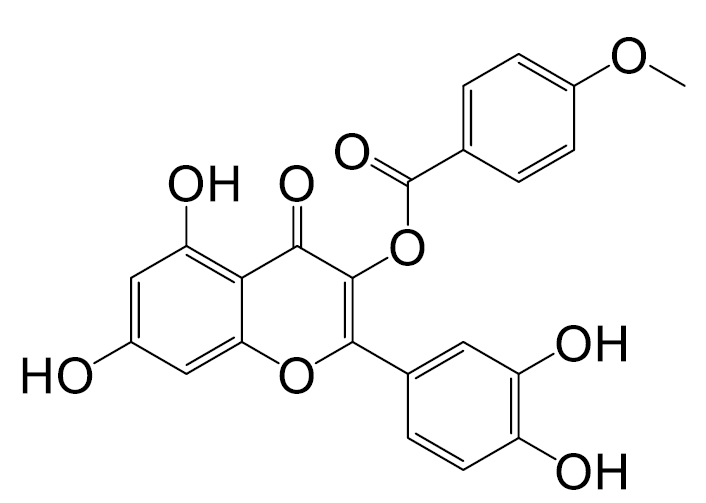  [2-(3,4-dihydroxyphenyl)-5,7-dihydroxy-4-oxochromen-3-yl] 4-methoxybenzoate  C_23_H_16_O_9_  129904993 | 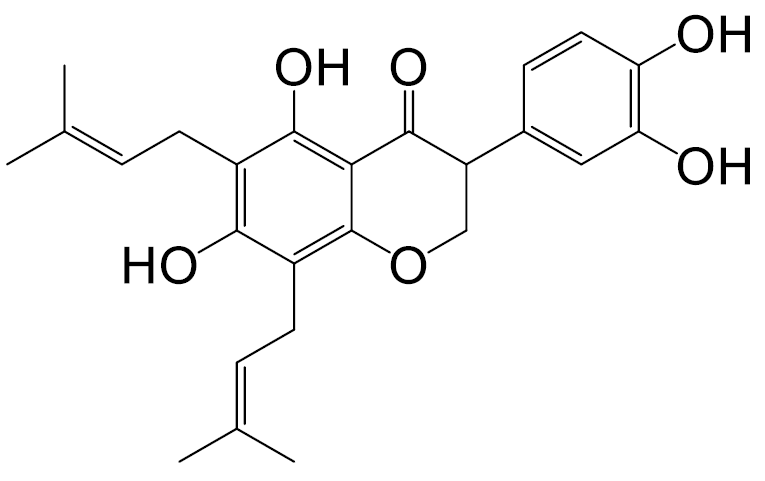  3-(3,4-dihydroxyphenyl)-5,7-dihydroxy-6,8-bis(3-methylbut-2-enyl)-2,3-dihydrochromen-4-one  C_25_H_28_O_6_  487089 |
| --- | --- | --- | --- | --- | --- |
| 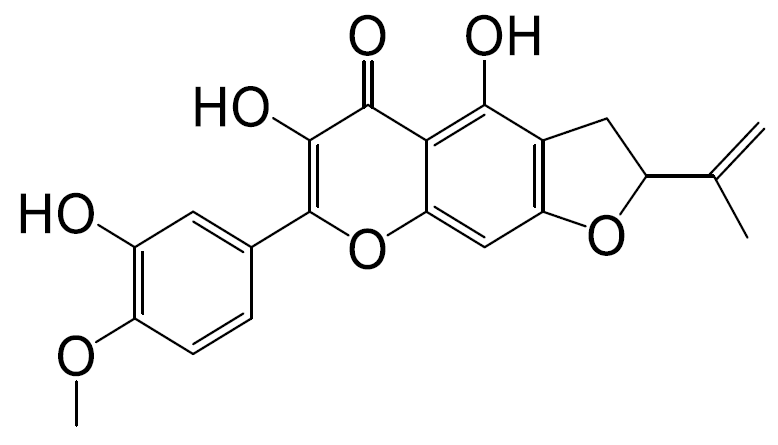  4,6-dihydroxy-7-(3-hydroxy-4-methoxyphenyl)-2-prop-1-en-2-yl-2,3-dihydrofuro[3,2-g]chromen-5-one  C_21_H_18_O_7_  15126638 | 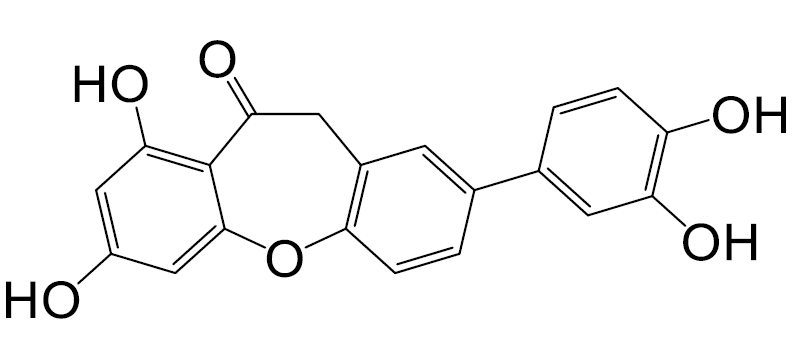  3-(3,4-dihydroxyphenyl)-7,9-dihydroxy-5*H*-benzo[b][1]benzoxepin-6-one  C_20_H_14_O_6_  20631775 | 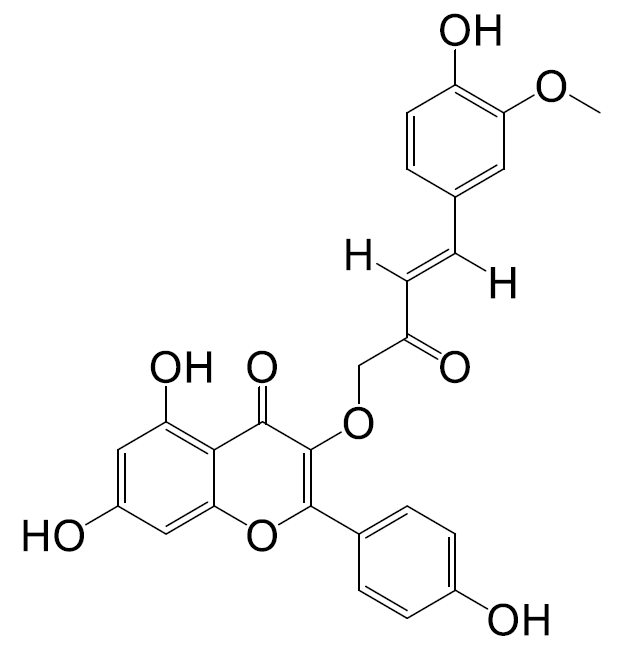  5,7-dihydroxy-3-[(*E*)-4-(4-hydroxy-3-methoxyphenyl)-2-oxobut-3-enoxy]-2-(4-hydroxyphenyl)chromen-4-one  C_26_H_20_O_9_  56641636 | 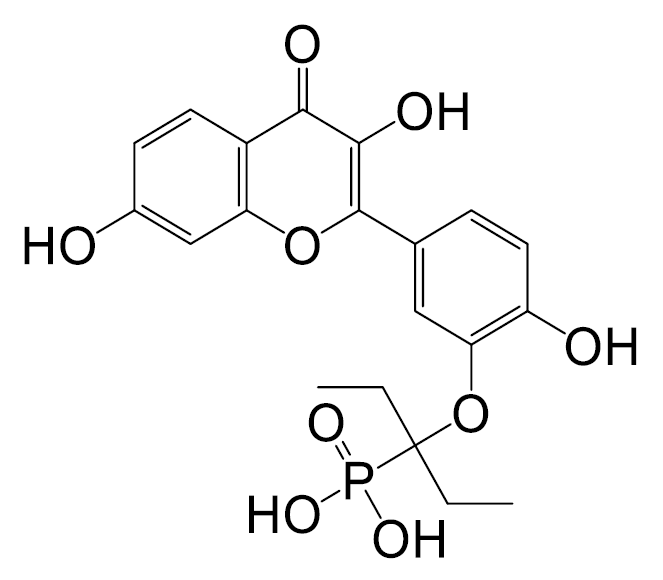  3-[5-(3,7-dihydroxy-4-oxochromen-2-yl)-2-hydroxyphenoxy]pentan-3-ylphosphonic acid  C_20_H_21_O_9_P 66600462 | 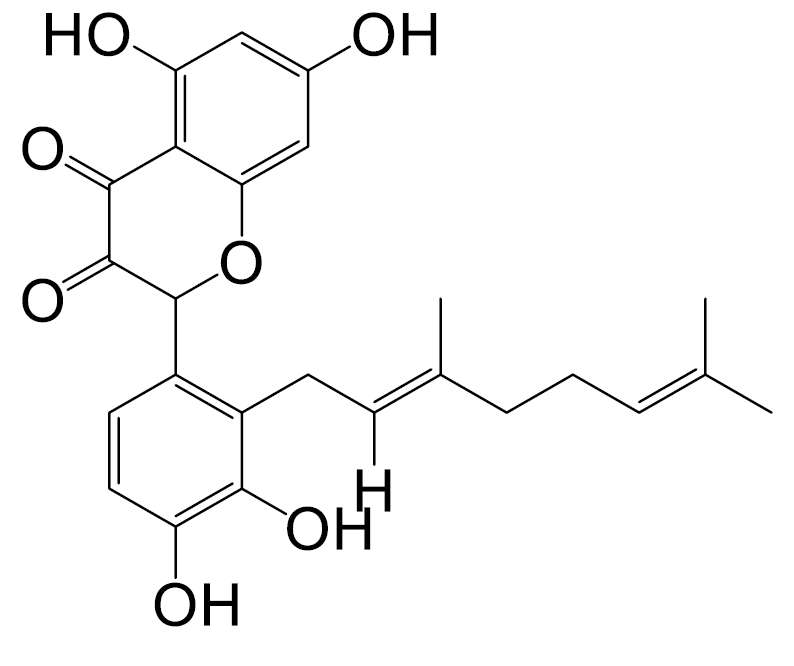  2-[2-[(2*E*)-3,7-dimethylocta-2,6-dienyl]-3,4-dihydroxyphenyl]-5,7-dihydroxychromene-3,4-dione  C_25_H_26_O_7_  71765672 | 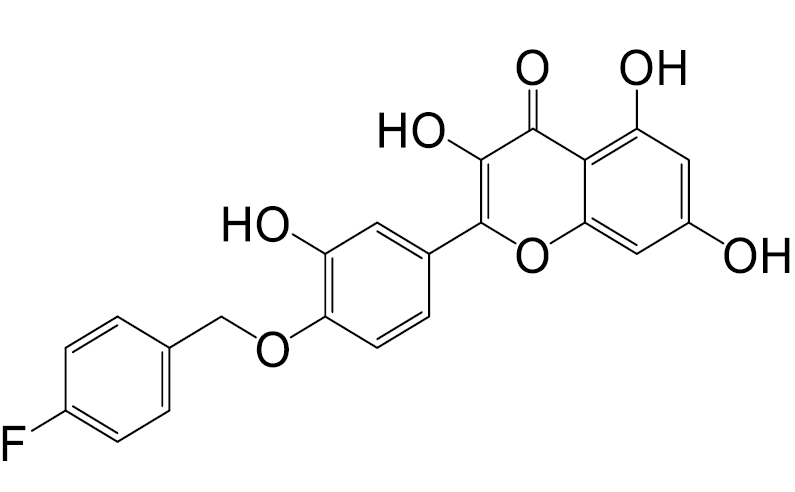  2-[4-[(4-fluorophenyl)methoxy]-3-hydroxyphenyl]-3,5,7-trihydroxychromen-4-one  C_22_H_15_FO_7_  90643987 |

Table S1 (continued) A list of 100 quercetin-like molecules with high docking score.

| 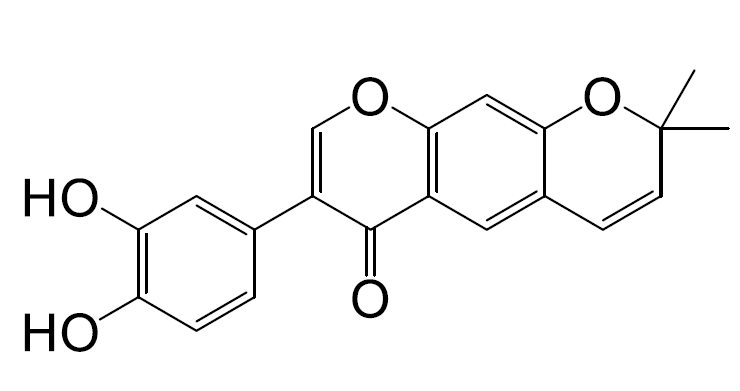  7-(3,4-dihydroxyphenyl)-2,2-dimethylpyrano[3,2-g]chromen-6-one  C_20_H_16_O_5_  101405688 | 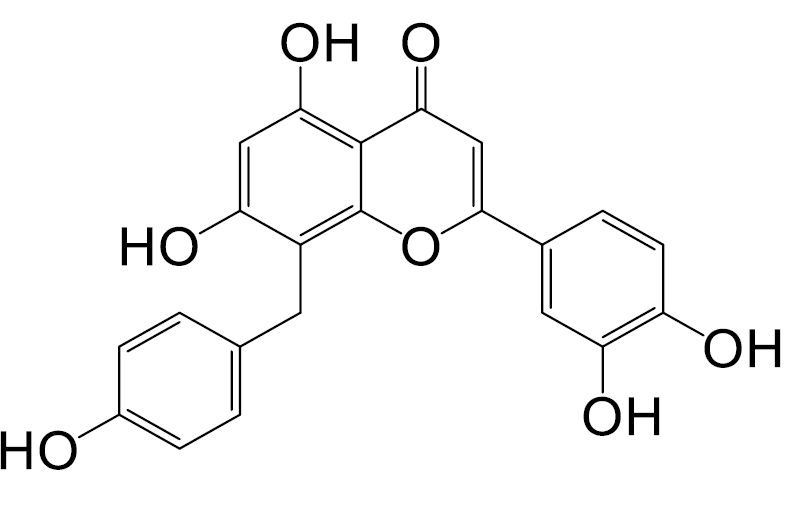  2-(3,4-dihydroxyphenyl)-5,7-dihydroxy-8-[(4-hydroxyphenyl)methyl]chromen-4-one  C_22_H_16_O_7_  101916306 | 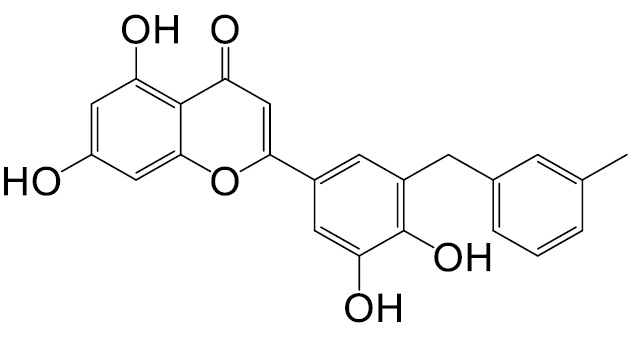  2-[3,4-dihydroxy-5-[(3-methylphenyl)methyl]phenyl]-5,7 dihydroxychromen-4-one  C_23_H_18_O_6_  127045150 | 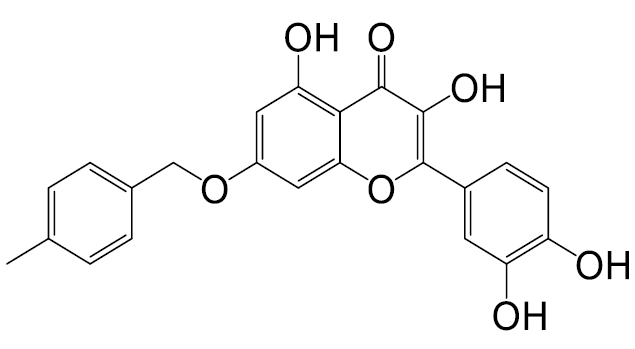  2-(3,4-dihydroxyphenyl)-3,5-dihydroxy-7-[(4-methylphenyl)methoxy]chromen-4-one  C_23_H_18_O_7_  129824763 | 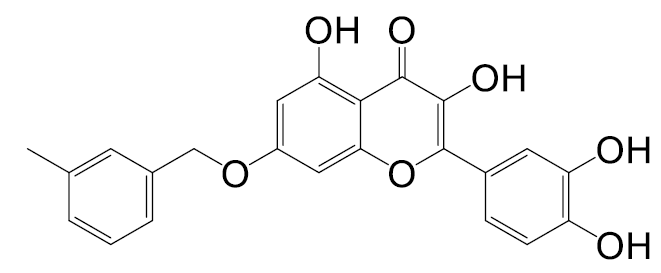  2-(3,4-dihydroxyphenyl)-3,5-dihydroxy-7-[(3-methylphenyl)methoxy]chromen-4-one  C_23_H_18_O_7_  129825792 | 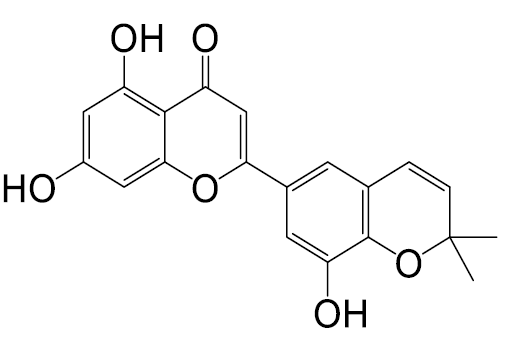  5,7-dihydroxy-2-(8-hydroxy-2,2-dimethylchromen-6-yl)chromen-4-one  C_20_H_16_O_6_  5315397 |
| --- | --- | --- | --- | --- | --- |
| 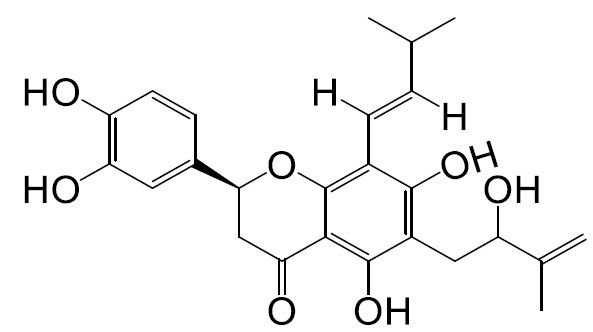  (2S)-2-(3,4-dihydroxyphenyl)-5,7-dihydroxy-6-(2-hydroxy-3-methylbut-3-enyl)-8-[(E)-3-methylbut-1-enyl]-2,3-dihydrochromen-4-one  C_25_H_28_O_7_  10575105 | 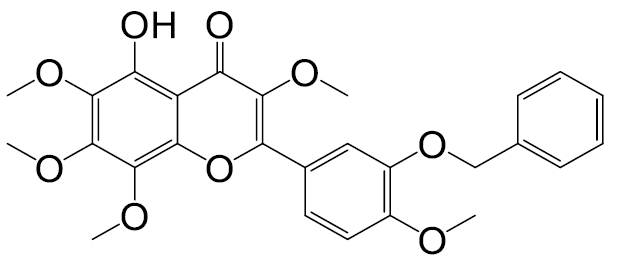  5-hydroxy-3,6,7,8-tetramethoxy-2-(4-methoxy-3-phenylmethoxyphenyl)chromen-4-one  C_27_H_26_O_9_  10577136 | 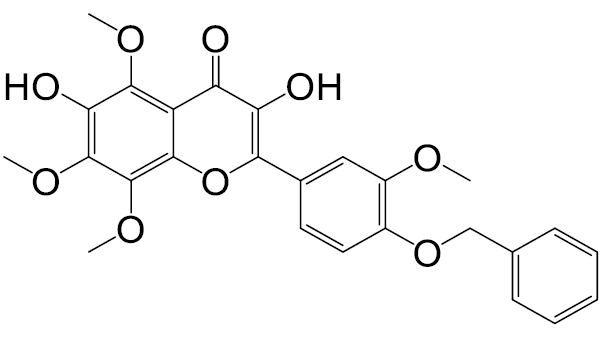  3,6-dihydroxy-5,7,8-trimethoxy-2-(3-methoxy-4-phenylmethoxyphenyl)chromen-4-one  C_26_H_24_O_9_  10648384 | 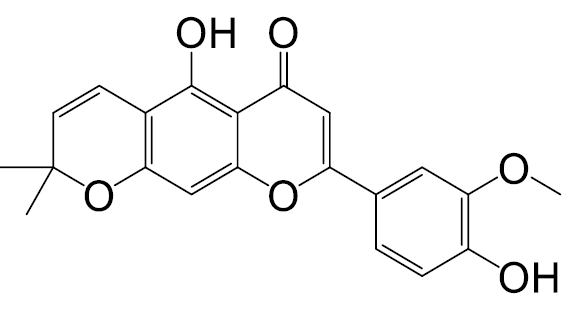  5-hydroxy-8-(4-hydroxy-3-methoxyphenyl)-2,2-dimethylpyrano[3,2-g]chromen-6-one  C_21_H_18_O_6_  10915521 | 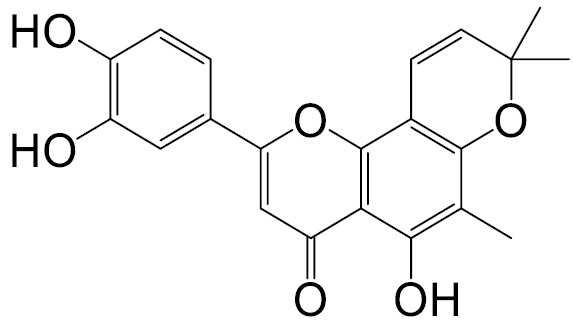  2-(3,4-dihydroxyphenyl)-5-hydroxy-6,8,8-trimethylpyrano[2,3-h]chromen-4-one  C_21_H_18_O_6_  11100628 | 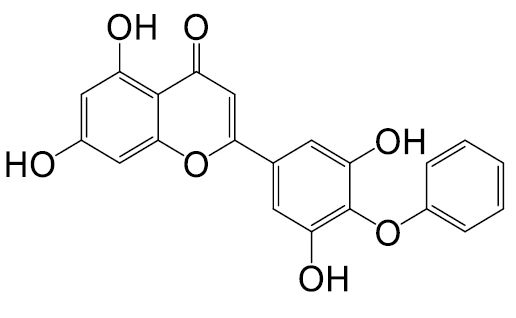  2-(3,5-dihydroxy-4-phenoxyphenyl)-5,7-dihydroxychromen-4-one  C_21_H_14_O_7_  20530145 |

Table S1 (continued) A list of 100 quercetin-like molecules with high docking score.

| 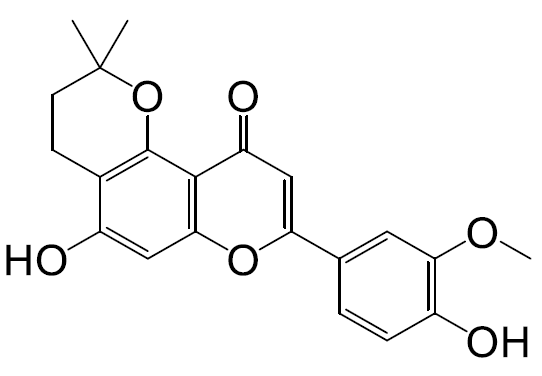  5-hydroxy-8-(4-hydroxy-3-methoxyphenyl)-2,2-dimethyl-3,4-dihydropyrano[2,3-f]chromen-10-one  C_21_H_20_O_6_  44258210 | 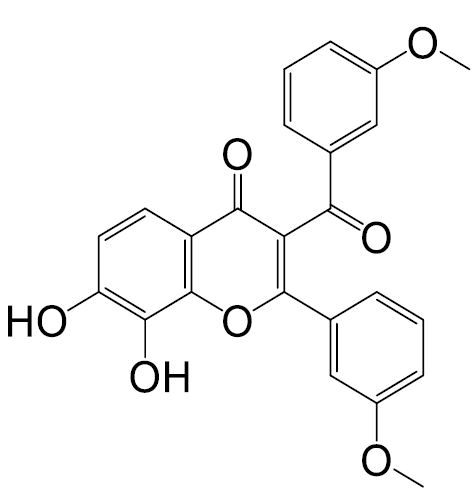  7,8-dihydroxy-3-(3-methoxybenzoyl)-2-(3-methoxyphenyl)chromen-4-one  C_24_H_18_O_7_  60165226 | 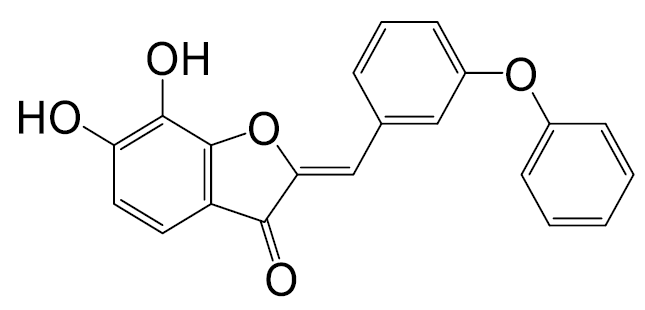  6,7-dihydroxy-2-[(3-phenoxyphenyl)methylidene]-1-benzofuran-3-one  C_21_H_14_O_5_  69838377 | 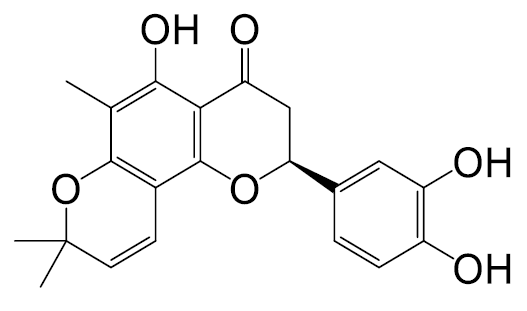  (2*S*)-2-(3,4-dihydroxyphenyl)-5-hydroxy-6,8,8-trimethyl-2,3-dihydropyrano[2,3-h]chromen-4-one  C_21_H_20_O_6_  71528833 | 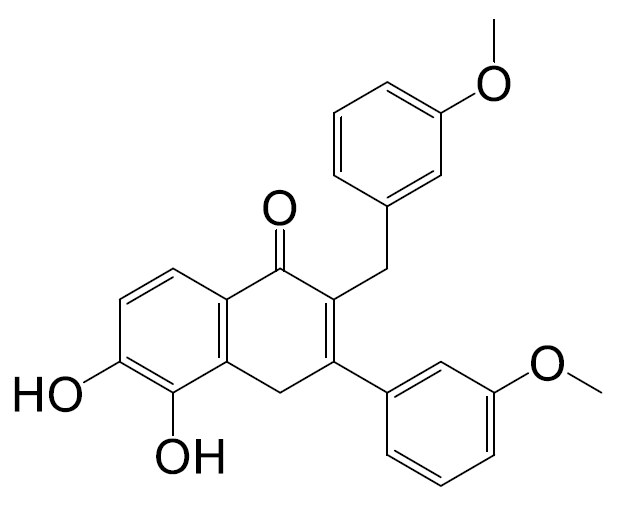  7,8-dihydroxy-2-(3-methoxyphenyl)-3-[(3-methoxyphenyl)methyl]chromen-4-one  C_24_H_20_O_6_  71592602 | 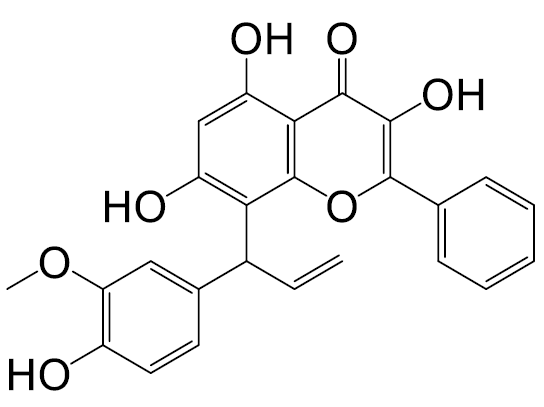  3,5,7-trihydroxy-8-[1-(4-hydroxy-3-methoxyphenyl)prop-2-enyl]-2-phenylchromen-4-one  C_25_H_20_O_7_  75149603 |
| --- | --- | --- | --- | --- | --- |
| 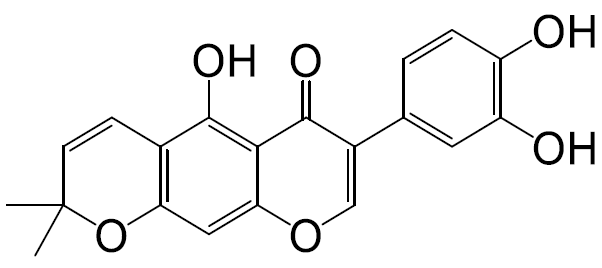  7-(3,4-dihydroxyphenyl)-5-hydroxy-2,2-dimethylpyrano[3,2-g]chromen-6-one  C_20_H_16_O_6_  76900284 | 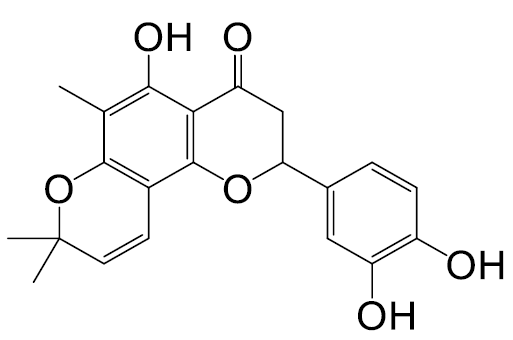  2-(3,4-dihydroxyphenyl)-5-hydroxy-6,8,8-trimethyl-2,3-dihydropyrano[2,3-h]chromen-4-one  C_21_H_20_O_6_  78099852 | 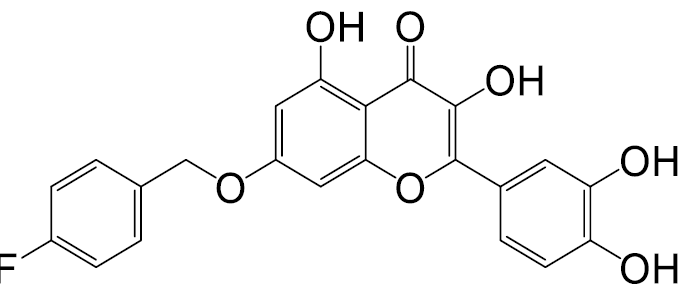  2-(3,4-dihydroxyphenyl)-7-[(4-fluorophenyl)methoxy]-3,5-dihydroxychromen-4-one  C_22_H_15_FO_7_  90643991 | 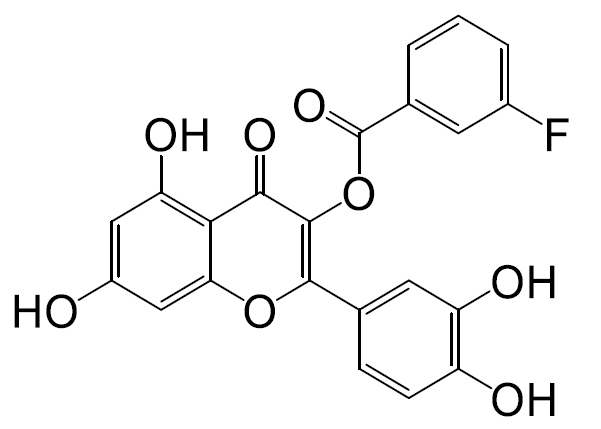  [2-(3,4-dihydroxyphenyl)-5,7-dihydroxy-4-oxochromen-3-yl] 3-fluorobenzoate  C_22_H_13_FO_8_  122230748 | 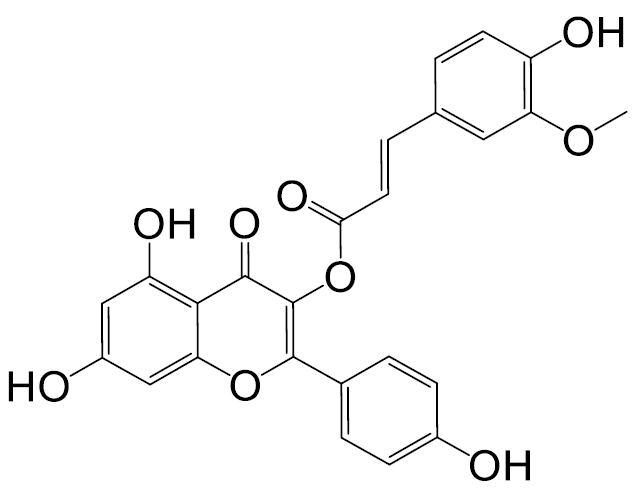  [5,7-dihydroxy-2-(4-hydroxyphenyl)-4-oxochromen-3-yl] 3-(4-hydroxy-3-methoxyphenyl)prop-2-enoate  C_25_H_18_O_9_  122421211 | 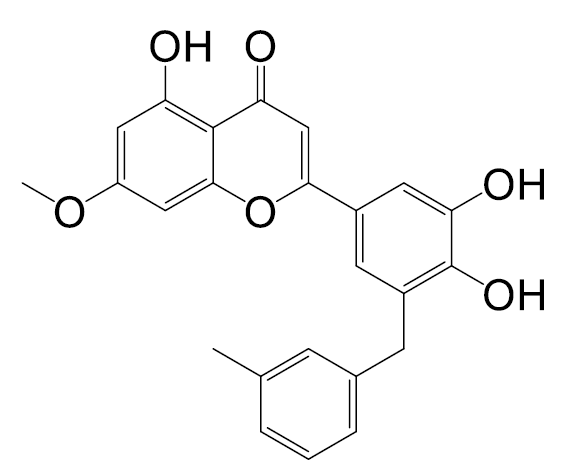  2-[3,4-dihydroxy-5-[(3-methylphenyl)methyl]phenyl]-5-hydroxy-7-methoxychromen-4-one  C_24_H_20_O_6_  127044660 |

Table S1 (continued) A list of 100 quercetin-like molecules with high docking score.

| 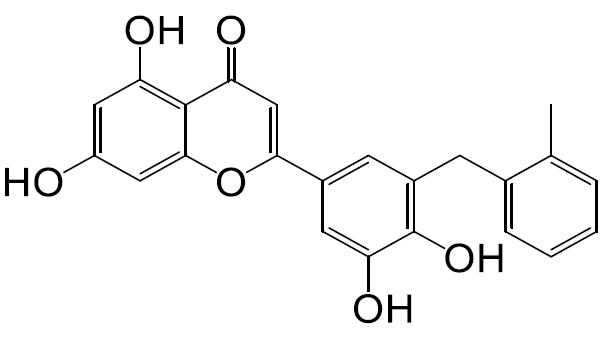  2-[3,4-dihydroxy-5-[(2-methylphenyl)methyl]phenyl]-5,7-dihydroxychromen-4-one  C_23_H_18_O_6_  127045149 | 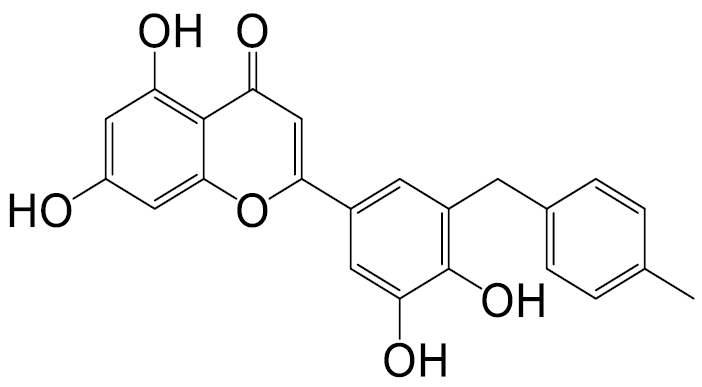  2-[3,4-dihydroxy-5-[(4-methylphenyl)methyl]phenyl]-5,7-dihydroxychromen-4-one  C_23_H_18_O_6_  127045295 | 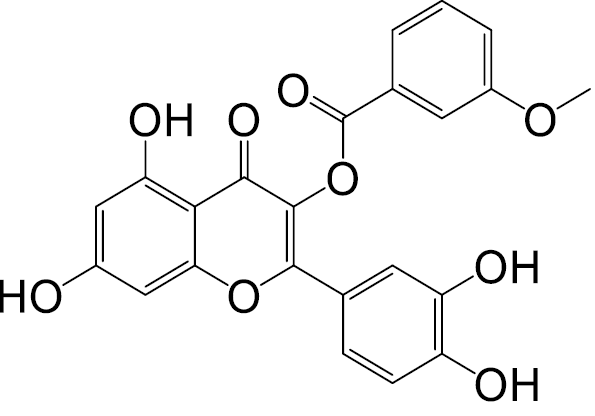  [2-(3,4-dihydroxyphenyl)-5,7-dihydroxy-4-oxochromen-3-yl] 3-methoxybenzoate  C_23_H_16_O_9_  129904780 | 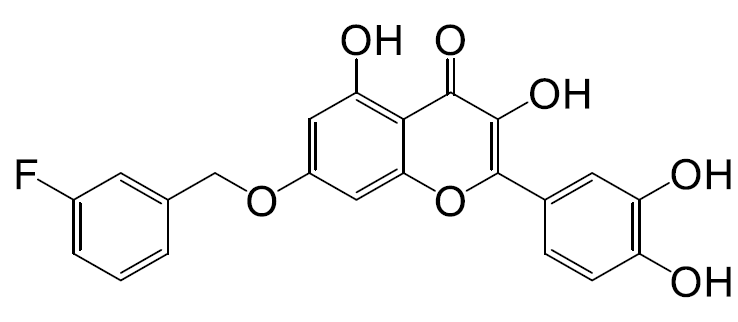  2-(3,4-dihydroxyphenyl)-7-[(3-fluorophenyl)methoxy]-3,5-dihydroxychromen-4-one  C_22_H_15_FO_7_  129904975 | 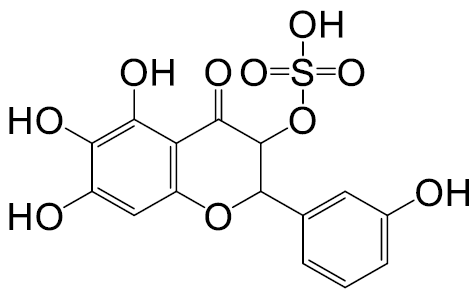  [5,6,7-trihydroxy-2-(3-hydroxyphenyl)-4-oxo-2,3-dihydrochromen-3-yl] hydrogen sulfate  C_15_H_12_O_10_S  131831857 | 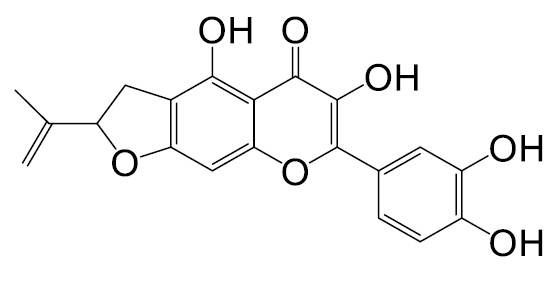  7-(3,4-dihydroxyphenyl)-4,6-dihydroxy-2-prop-1-en-2-yl-2,3-dihydrofuro[3,2-g]chromen-5-one  C_20_H_16_O_7_  442663 |
| --- | --- | --- | --- | --- | --- |
| 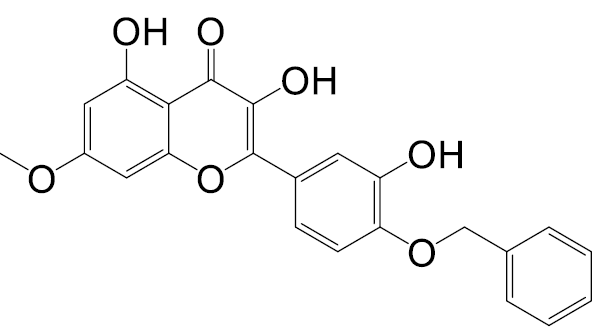  3,5-dihydroxy-2-(3-hydroxy-4-phenylmethoxyphenyl)-7-methoxychromen-4-one  C_23_H_18_O_7_  5704578 | 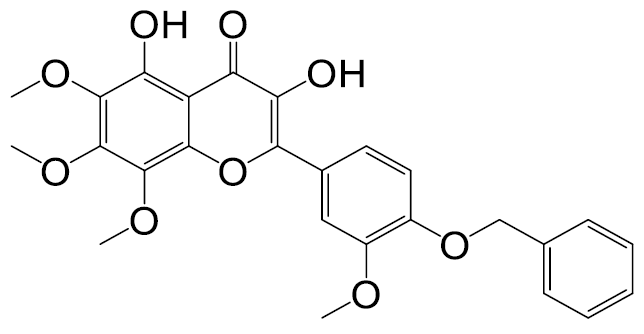  3,5-dihydroxy-6,7,8-trimethoxy-2-(3-methoxy-4-phenylmethoxyphenyl)chromen-4-one  C_26_H_24_O_9_  10528717 | 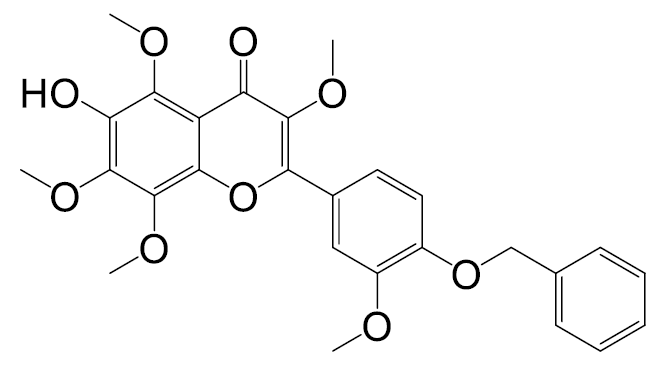  6-hydroxy-3,5,7,8-tetramethoxy-2-(3-methoxy-4-phenylmethoxyphenyl)chromen-4-one  C_27_H_26_O_9_  10529220 | 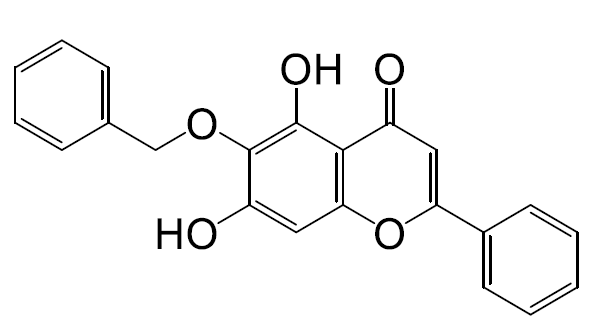  5,7-dihydroxy-2-phenyl-6-phenylmethoxychromen-4-one  C_22_H_16_O_5_  11314387 | 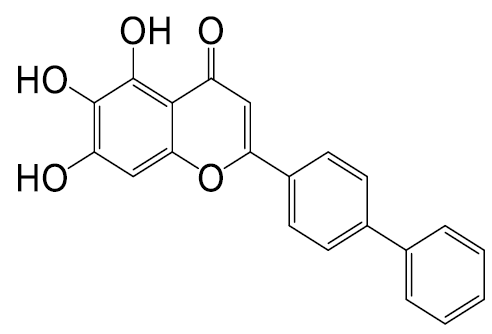  5,6,7-trihydroxy-2-(4-phenylphenyl)chromen-4-one  C_21_H_14_O_5_  11416700 | 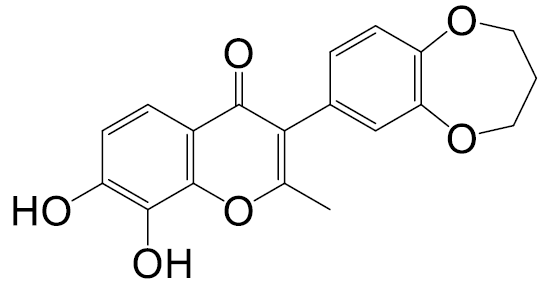  3-(3,4-dihydro-2*H*-1,5-benzodioxepin-7-yl)-7,8-dihydroxy-2-methylchromen-4-one  C_19_H_16_O_6_  15658884 |

Table S1 (continued) A list of 100 quercetin-like molecules with high docking score.

| 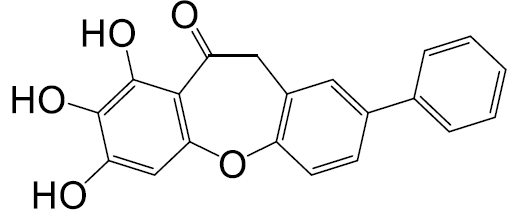  2,3,4-trihydroxy-8-phenyl-6*H*-benzo[b][1]benzoxepin-5-one  C_20_H_14_O_5_  20631776 | 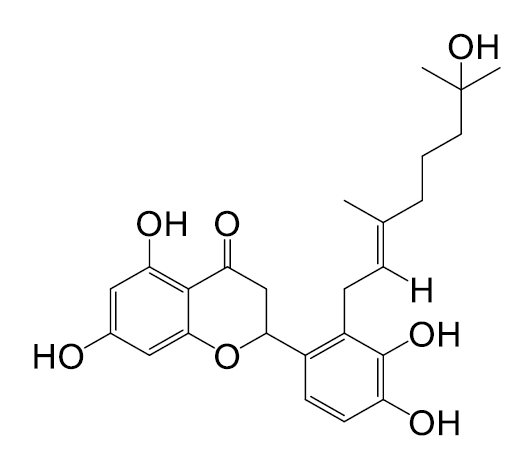  2-[3,4-dihydroxy-2-[(*E*)-7-hydroxy-3,7-dimethyloct-2-enyl]phenyl]-5,7-dihydroxy-2,3-dihydrochromen-4-one  C_25_H_30_O_7_  21629595 | 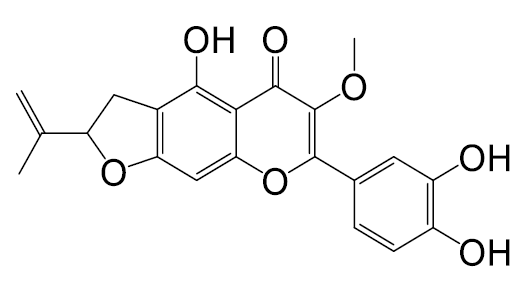  7-(3,4-dihydroxyphenyl)-4-hydroxy-6-methoxy-2-prop-1-en-2-yl-2,3-dihydrofuro[3,2-g]chromen-5-one  C_21_H_18_O_7_  44259674 | 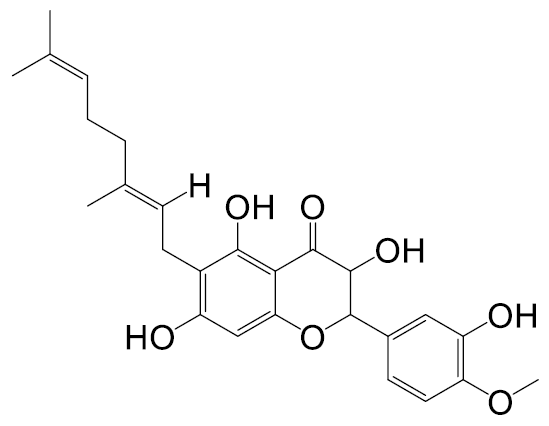  6-[(2E)-3,7-dimethylocta-2,6-dienyl]-3,5,7-trihydroxy-2-(3-hydroxy-4-methoxyphenyl)-2,3-dihydrochromen-4-one  C_26_H_30_O_7_  57520492 | 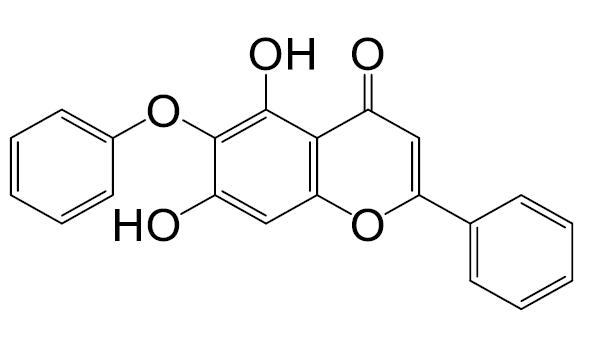  5,7-dihydroxy-6-phenoxy-2-phenylchromen-4-one  C_21_H_14_O_5_  66862728 | 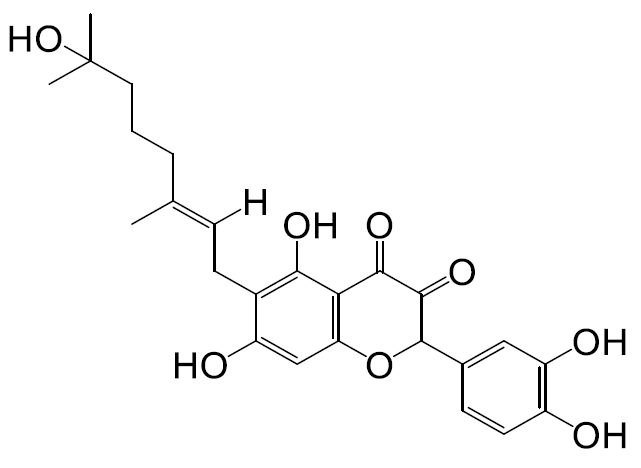  3-(3,4-dihydro-2*H*-1,5-benzodioxepin-7-yl)-7,8-dihydroxy-2-methylchromen-4-one  C_19_H_16_O_6_  15658884 |
| --- | --- | --- | --- | --- | --- |
| 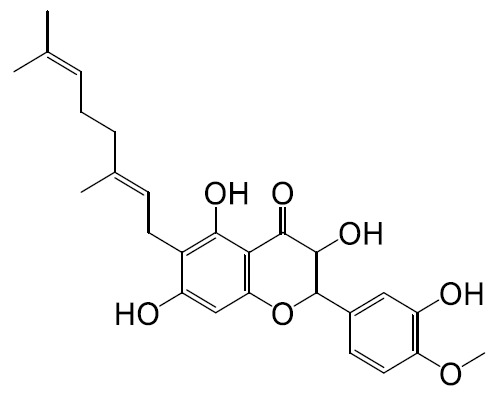  6-(3,7-dimethylocta-2,6-dienyl)-3,5,7-trihydroxy-2-(3-hydroxy-4-methoxyphenyl)-2,3-dihydrochromen-4-one  C_26_H_30_O_7_  74333957 | 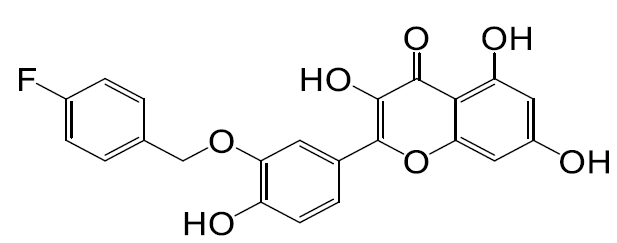  2-[3-[(4-Fluorophenyl)methoxy]-4-hydroxyphenyl]-3,5,7-trihydroxychromen-4-one  C_22_H_15_FO_7_  90643986 | 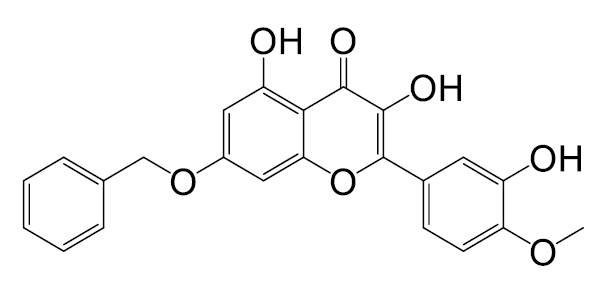  3,5-dihydroxy-2-(3-hydroxy-4-methoxyphenyl)-7-phenylmethoxychromen-4-one  C_23_H_18_O_7_  102595093 | 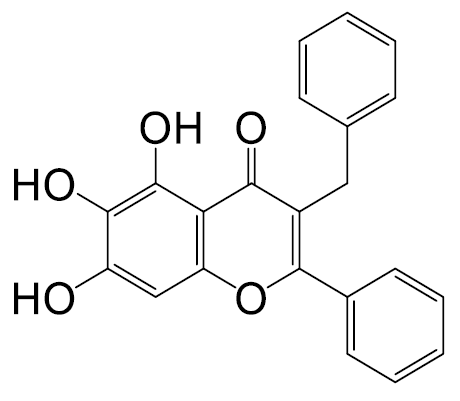  3-benzyl-5,6,7-trihydroxy-2-phenylchromen-4-one  C_22_H_16_O_5_  123375132 | 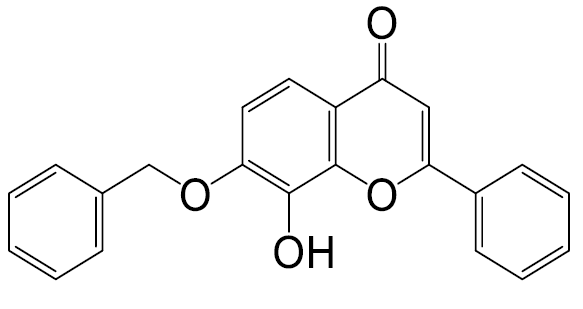  8-hydroxy-2-phenyl-7-phenylmethoxychromen-4-one  C_22_H_16_O_4_  129825136 | 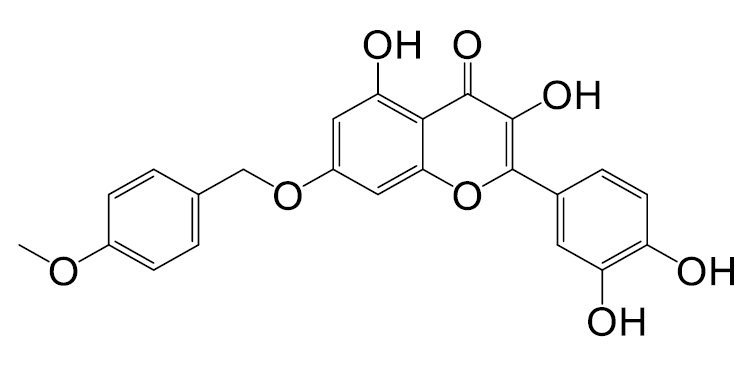  2-(3,4-dihydroxyphenyl)-3,5-dihydroxy-7-[(4-methoxyphenyl)methoxy]chromen-4-one  C_23_H_18_O_8_  129825762 |

Table S1 (continued) A list of 100 quercetin-like molecules with high docking score.

| 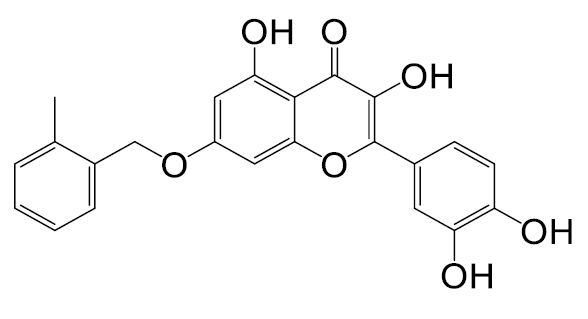  2-(3,4-dihydroxyphenyl)-3,5-dihydroxy-7-[(2-methylphenyl)methoxy]chromen-4-one  C_23_H_18_O_7_  129825788 | 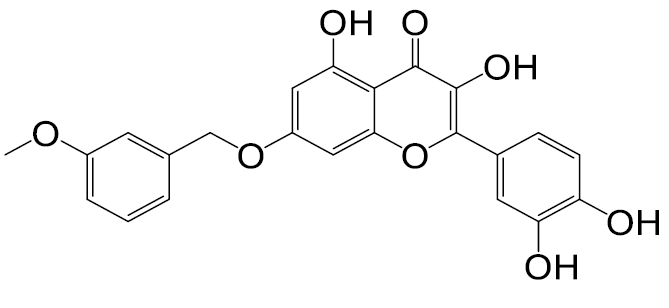  2-(3,4-dihydroxyphenyl)-3,5-dihydroxy-7-[(3-methoxyphenyl)methoxy]chromen-4-one  C_23_H_18_O_8_  129825797 | 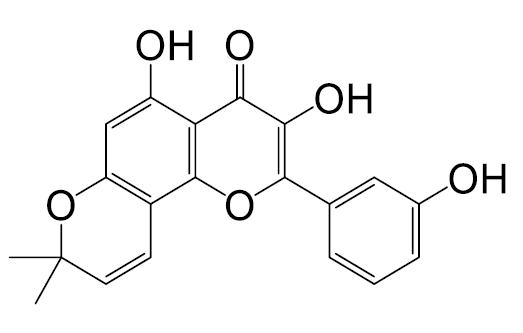  3,5-dihydroxy-2-(3-hydroxyphenyl)-8,8-dimethylpyrano[2,3-h]chromen-4-one  C_20_H_16_O_6_  131833748 | 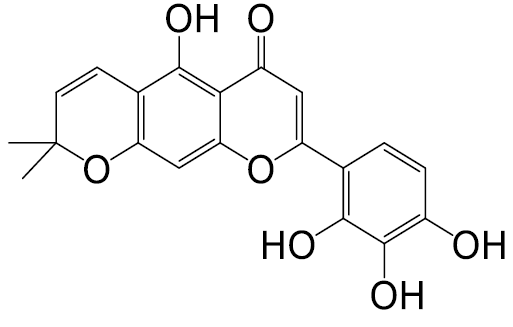  5-hydroxy-2,2-dimethyl-8-(2,3,4-trihydroxyphenyl)pyrano[3,2-g]chromen-6-one  C_20_H_16_O_7_  131833783 |  |  |
| --- | --- | --- | --- | --- | --- |

Table S2 A List of considered COMPs with pharmaceutical activity selected by Lipinski’s rule of five.

| **COMP** | **IUPAC name** | **Molecular formula** | **Molecular weight** | **#Atoms** | **logP** | **#HBD** | **#HBA** | **Chemical Structure** |
| --- | --- | --- | --- | --- | --- | --- | --- | --- |
| **1** | 5,7-dihydroxy-2-(4-hydroxy-3,5-dimethoxyphenyl)-3-phenylchromen-4-one | C_23_H_18_O_7_ | 406.3848 | 48 | 4.2610 | 3 | 7 |  |
| **2** | 3-(3,4-dihydroxyphenyl)-5-hydroxy-6-(2-hydroxy-3-methylbut-3-enyl)-8,8-dimethylpyrano[2,3-h]chromen-4-one | C_25_H_24_O_7_ | 436.4539 | 56 | 4.2405 | 4 | 7 |  |
| **3** | (4E)-3-(3,4-dihydroxyphenyl)-4-[(3,4-dihydroxyphenyl)methylidene]-8-hydroxy-2,3-dihydroxanthene-1,9-dione | C_26_H_18_O_8_ | 458.4163 | 52 | 4.2318 | 5 | 8 |  |
| **4** | (2S)-2-[3,4-dihydroxy-2-[(Z)-7-hydroxy-3,7-dimethyloct-2-enyl]phenyl]-5,7-dihydroxy-2,3-dihydrochromen-4-one | C25H30O7 | 442.5015 | 62 | 4.6455 | 5 | 7 |  |
| **5** | 7-cyclohexyl-3-hydroxy-2-(3,4,5-trihydroxyphenyl)chromen-4-one | C21H20O6 | 368.3799 | 47 | 4.3301 | 4 | 6 |  |

Table S2 (continued) A List of considered COMPs with pharmaceutical activity selected by Lipinski’s rule of five.

| **COMP** | **IUPAC name** | **Molecular formula** | **Molecular weight** | **#Atoms** | **logP** | **#HBD** | **#HBA** | **Chemical Structure** |
| --- | --- | --- | --- | --- | --- | --- | --- | --- |

| **6** | 3,5,7-trihydroxy-2-(8-hydroxy-2,2-dimethylchromen-6-yl)-8-(2-methylbut-3-en-2-yl)chromen-4-one | | | C_25_H_24_O_7_ | | 436.4539 | | 56 | | 4.9302 | | 4 | | 7 | |  | |
| --- | --- | --- | --- | --- | --- | --- | --- | --- | --- | --- | --- | --- | --- | --- | --- | --- | --- |
| **7** | | 5,6,7-trihydroxy-2,3-diphenylchromen-4-one | C_21_H_14_O_5_ | | 346.3329 | | 40 | | 4.2438 | | 3 | | 5 | |  | |  |
| **8** | | (3R)-6-[(2E)-3,7-dimethylocta-2,6-dienyl]-3,5,7-trihydroxy-2-(3-hydroxy-4-methoxyphenyl)-2,3-dihydrochromen-4-one | C_26_H_30_O_7_ | | 454.5122 | | 63 | | 4.7245 | | 4 | | 7 | |  | |  |
| **9** | | 1,3,4,8-tetrahydroxy-2,7-bis(3-methylbut-2-enyl)xanthen-9-one | C_23_H_24_O_6_ | | 396.4331 | | 53 | | 4.786 | | 4 | | 6 | |  | |  |
| **10** | | [4-(5,7-dihydroxy-4-oxochromen-2-yl)-2-hydroxyphenyl] hydrogen sulfate | C_15_H_10_O_9_S | | 366.2995 | | 35 | | 2.8392 | | 4 | | 9 | |  | |  |

Table S2 (continued) A List of considered COMPs with pharmaceutical activity selected by Lipinski’s rule of five.

| **COMP** | **IUPAC name** | **Molecular**  **formula** | **Molecular**  **weight** | **#Atoms** | **logP** | **#HBD** | **#HBA** | **Chemical Structure** |
| --- | --- | --- | --- | --- | --- | --- | --- | --- |
| **11** | 5-hydroxy-2-(8-hydroxy-2,2-dimethylchromen-6-yl)-7-methoxy-6,8-dimethylchromen-4-one | C_23_H_22_O_6_ | 394.4172 | 51 | 4.6808 | 2 | 6 |  |
| **12** | 3-hydroxy-2-phenyl-5-(3,4,5-trihydroxybenzoyl)chromen-4-one | C_22_H_14_O_7_ | 390.3424 | 43 | 3.5134 | 4 | 7 |  |
| **13** | 5-hydroxy-2,2-dimethyl-8-[2,4,5-trihydroxy-3-(3-methylbut-2-enyl)phenyl]pyrano[3,2-g]chromen-6-one | C_25_H_24_O_7_ | 436.4539 | 56 | 4.9753 | 4 | 7 |  |
| **14** | 2-[3,4-dihydroxy-5-[(E)-6-hydroxy-3,6-dimethylhept-2-enyl]phenyl]-5,7-dihydroxy-2,3-dihydrochromen-4-one | C_24_H_28_O_7_ | 428.4749 | 59 | 4.2554 | 5 | 7 |  |
| **15** | (2S)-2-[3,4-dihydroxy-5-[(Z)-7-hydroxy-3,7-dimethyloct-2-enyl]phenyl]-5,7-dihydroxy-2,3-dihydrochromen-4-one | C_25_H_30_O_7_ | 442.5015 | 62 | 4.6455 | 3 | 7 |  |
| quercetin | 2-(3,4-dihydroxyphenyl)-3,5,7-trihydroxychromen-4-one | C_15_H_10_O_7_ | 302.2357 | 32 | 1.988 | 5 | 7 |  |

**Figure S1**

Comparison of *φ/ψ* desity of obatained structural information from 40 ns MD simulation of fPrP_170-229_ decamers from three independent replicas in explicit solvent model or in implicit solvent model, respectively. Yellow squares represent *φ/ψ* from residues of fPrP_170-229._

**Figure S2**

Docking pose of COMPs **1**-**5** with fPrP_170-229_. The binding cavity is shown in an enlarged frame.

**Figure S3**

The interaction network of COMPs **1-5** with fPrP_170-229_. The alphabets in the parentheses represent fibril chains in which the corresponding residues locate. The backbone of quercetin is shown in purple. The residues playing the role of hydrogen bond donors are labeled in green. The residues interacting with quercetin through van der Waals interaction are labeled in black with red spoked arcs.

**Figure S4**

The final structures of COMP-fPrP_170-229_ complexes after 162 ns MD simulation.

**Figure S5**

RMSD of fPrP_170-229_ backbone. (**A**) COMP **1** model A-C, (**B**) COMP **2** model A-C, (**C**) COMP **3** model A-C, (**D**) COMP **4** model A-C, and (**E**) COMP **5** model A-C.

**Figure S6**

Binding energy of COMP **1**-**5** with fibril in MM/GBSA model.

**Figure S7**

RMSF of COMP-fPrP_170-229_ complexes.

**Figure S8**

The secondary structure of treated fPrP_170-229_ from the last 20 ns trajectory.

**Figure S9**

The alignment of fPrP_170-229_ and 263K PrP^Sc^ from residue 170 to 227. The fPrP_170-229_, 263K PrP^Sc^ and two glycosylation sites are label with blue, green, black, and red, respectively.

**Figure S10**

The structure of mutated PrP E196K fPrP_175-217_. (A) fPrP_175-217_ structure, (B) the alignment of fPrP_170-229_ and fPrP_175-217_. The fPrP_170-229_ and fPrP_175-217_ are label with blue and green, respectively.
